# Engineered Extracellular Vesicles Modified by Angiopep-2 Peptide Promote Targeted Repair of Spinal Cord Injury and Brain Inflammation

**DOI:** 10.1021/acsnano.4c14675

**Published:** 2025-01-24

**Authors:** Guang Kong, Jie Liu, Juan Wang, Xiaohu Yu, Cong Li, Mingyang Deng, Minhao Liu, Siming Wang, Chunming Tang, Wu Xiong, Jin Fan

**Affiliations:** † Department of Orthopedics, The First Affiliated Hospital of Nanjing Medical University, 300 Guangzhou Road, Nanjing 210000 Jiangsu, China; ‡ Department of Orthopedics, Xijing Hospital, Fourth Military Medical University, Xi’an 710000 Shaanxi, China; § Department of Orthopedics, The Affiliated Taizhou People’s Hospital of Nanjing Medical University, Taizhou School of Clinical Medicine, Nanjing Medical University, 366 Taihu Road, Taizhou 225300 Jiangsu, China; ∥ Department of Human Anatomy, School of Basic Medicine, Nanjing Medical University, Nanjing 210000 Jiangsu, China; ⊥ Department of Pharmaceutics, School of Pharmacy, Nanjing Medical University, 300 Guangzhou Road, Nanjing 210000 Jiangsu, China

**Keywords:** engineered extracellular vesicles, angiopep-2, melatonin, spinal cord injury, cerebral inflammation, axonal regeneration

## Abstract

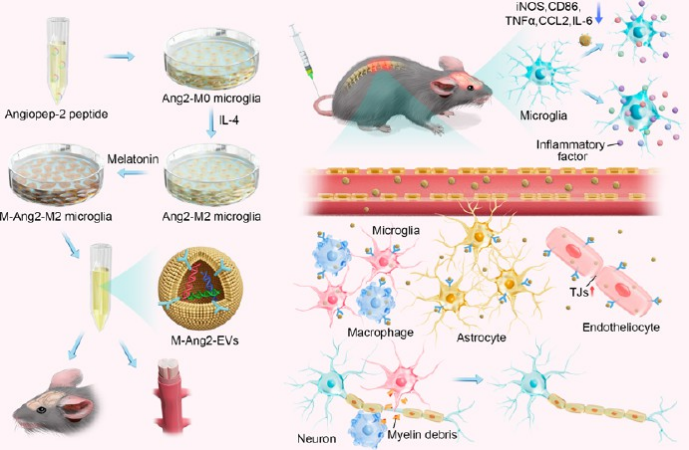

Engineered extracellular vesicles play an increasingly important role in the treatment of spinal cord injury. In order to prepare more effective engineered extracellular vesicles, we biologically modified M2 microglia. Angiopep-2 (Ang2) is an oligopeptide that can target the blood–brain barrier. Through single-cell sequencing and immunofluorescence experiments, we confirmed that the expression of LRP-1, the targeted receptor of Ang2, was elevated after spinal cord injury. Subsequently, we integrated the Ang2 peptide segment into M2 microglia to obtain Ang2-EVs, which could successfully target the site of spinal cord injury. However, in order to improve the function of Ang2-EVs, we pretreated M2 microglia with melatonin, which has anti-inflammatory effects, to obtain M-Ang2-EVs. The results of single-nucleus sequencing of the mouse spinal cord verified that neurons and OPCs gradually transformed into subtypes related to nerve repair functions after treatment with M-Ang2-EVs. This is consistent with the sequencing and enrichment analysis of miRNAs contained in M-Ang2-EVs. We further verified through experiments that M-Ang2-EVs can promote microglia/macrophages to phagocytose sphingomyelin, promote axon remyelination and axon elongation, and maintain the integrity of the blood-spinal barrier. Since Ang2 can also target the blood–brain barrier, we found that M-Ang2-EVs can also reduce brain inflammation that results from spinal cord injury. Our study applied the Angiopep-2 peptide to spinal cord injury to enhance the targeting of injured cells, and successfully construct engineered extracellular vesicles that can target the spinal cord injury site and the brain.

## Introduction

Spinal cord injury (SCI) is a devastating condition with poor prognosis, characterized by primary and secondary injury phases.^1^ The primary injury leads to the disruption of tissue structure and the blood-spinal cord barrier at the injury site, resulting in cell death and the release of various inflammatory factors and intracellular substances.^2^ These events subsequently trigger secondary injury processes. Secondary injury mainly involves pathological processes such as ischemia and hypoxia, neuroinflammation, oxidative stress, and cellular edema, all of which significantly impede tissue repair and motor function recovery following SCI.^3^ Since primary injury cannot be prevented, understanding the pathophysiological mechanisms of secondary injury is crucial for the development of effective treatments for SCI.

However, due to the presence of the blood-spinal cord barrier, many therapeutic cells and drugs cannot reach the site of spinal cord injury through the vasculature, imposing significant limitations on SCI treatment. Consequently, biologically derived extracellular vesicles (EVs) that can cross the blood-spinal cord barrier have garnered considerable attention in the context of SCI therapy.^4,5^ EVs are a heterogeneous group of cell-derived membrane structures that contain numerous bioactive components. They play crucial roles in mediating cell communication, regulating tissue repair, and immune surveillance.^6^ Increasing evidence suggests that EVs hold promise for improving drug delivery and therapeutic outcomes due to their high biocompatibility, low cytotoxicity, and immune inertness.^7,8^ Similarly, EVs play an increasingly important role in the treatment of spinal cord injury, and it is particularly important to select a precise cell to obtain therapeutic EVs.

Microglia are the resident immune cells of the central nervous system (CNS) and undergo heterogeneous activation in response to neurological diseases.^9^ The activation morphology of microglia can be divided into two opposite types, M1 and M2, and depending on the activated phenotype, microglia can produce cytotoxic or neuroprotective effects.^10−12^ M1 microglia initially respond to injury and infection but can also induce neurotoxicity and often establish a vicious cycle between neuronal death and acute inflammation, leading to more severe damage.^13,14^ In contrast, M2 microglia are the primary effector cells with the potential to inhibit pro-inflammatory immune responses and promote the expression of repair genes, aiding in the resolution of inflammation and promoting neuronal survival.^15^ Recent studies have demonstrated that exosomes derived from M2 microglia can alleviate secondary injury by activating A1 astrocytes, and other studies have indicated that these exosomes can promote neural recovery following spinal cord injury, thus offering a therapeutic strategy for SCI.^16,17^ Because of the therapeutic effect of M2 microglia, we used them as donor cells of extracellular vesicles. Research has shown that the targeted modification of EVs using biological or chemical methods can enhance their ability to aggregate around receptor cells, thereby improving delivery efficiency and therapeutic efficacy.^18,19^ These engineered EVs have been widely applied in the treatment of various diseases. To enhance the therapeutic efficacy of EVs, we engineered extracellular vesicles to target the site of spinal cord injury. Angiopep-2 (Ang2) is a 19-amino-acid-long oligopeptide derived from the Kunitz protease inhibitor domain, which effectively targets the low-density lipoprotein receptor-related protein-1 (LRP-1) in the blood–brain barrier, has been widely studied as a targeted therapy for brain diseases.^20,21^ While the targeting ability of Ang2 in spinal cord and spinal cord injury is not yet well understood, we conducted single-cell analysis and immunofluorescence staining on spinal cord tissue and found that LRP-1 is expressed in several key cell types within the spinal cord. This suggests the potential for Ang2 to target cells in the spinal cord. Therefore, we integrated Ang2 peptide into microglia and stimulated microglia to the M2 phenotype to obtain Ang2-EVs, which can effectively target the site of spinal cord injury. This modification represents a significant improvement and offers a novel approach to the treatment of spinal cord injury.

Increasing research indicates that preconditioning donor cells with drugs can enhance their functionality, thereby producing EVs with improved biological functions.^22,23^ This preconditioning method is gaining attention for its potential in optimizing EVs and treating various diseases. Melatonin, an indoleamine hormone with anti-inflammatory, antioxidant, and free radical scavenging properties, has shown protective effects in liver injury and brain injury.^24−26^ Therefore, we hypothesize that melatonin may also have beneficial effects in the treatment of SCI.

To enhance the biological functions of Ang2-EVs, we pretreated M2 microglia with melatonin, generating melatonin-preconditioned M2 microglia-derived extracellular vesicles (M-Ang2-EVs). Subsequent sequencing analysis of the miRNAs within these vesicles revealed that M-Ang2-EVs enhance microglial phagocytosis of myelin, promote axonal remyelination and extension, and regulate tight junction functionality, thereby reducing the permeability of the blood-spinal cord barrier(BSCB). These findings suggest that our engineered EVs represent a functional advancement in the treatment of SCI.

In this study, we integrated the Ang2 peptide into M2 microglia and pretreated these cells with melatonin to obtain M-Ang2-EVs. These engineered EVs not only leverage their targeting ability to increase the concentration of EVs at the site of spinal cord injury but also utilize the biological properties of melatonin to enhance the functionality of individual vesicles. This dual approach improves the efficiency of M2-EVs in treating spinal cord injury both in terms of quantity and quality.

## Results

### Elevated Expression of LRP-1 after Spinal Cord Injury

**Figure 1:**
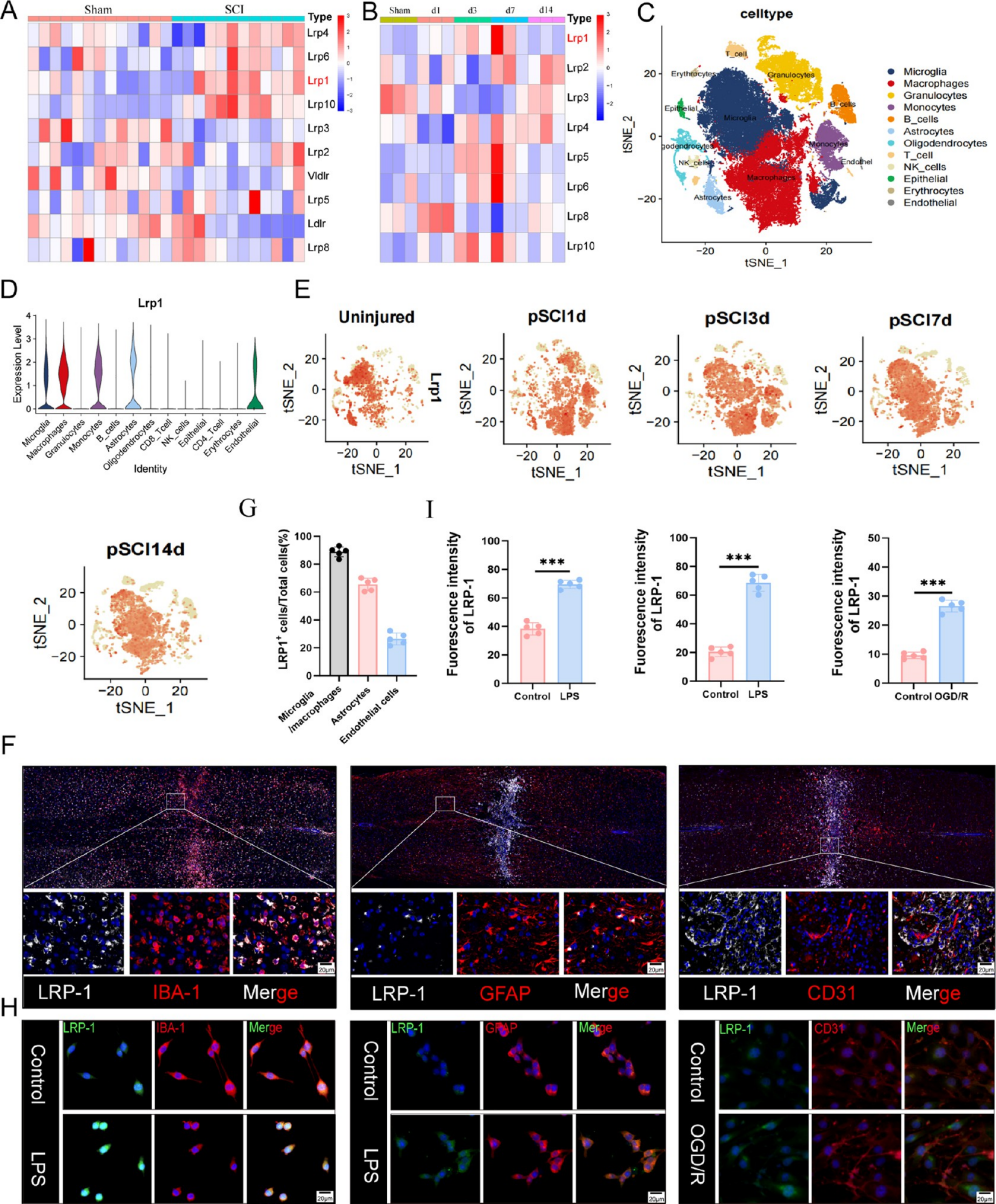
Expression of LRP-1 increased after spinal cord injury. (A) Heatmaps revealed the expression of LRP-1 in Sham group and SCI group. (B) Heatmaps revealed the expression of LRP-1 in spinal cord at different time points after injury. (C) t-Distributed Stochastic Neighbor Embedding (tSNE) shows the distribution of various cell populations after spinal cord injury. (D) The distribution of LRP-1 in various cell populations after spinal cord injury. (E) tSNE reveals the expression of LRP-1 in different cells at various time points after spinal cord injury. (F,G) Immunofluorescence staining after injury shows the expression of LRP-1 in microglia/macrophages, astrocytes, and endothelial cells. LRP-1 (white), IBA-1 labeled microglia/macrophages, GFAP labeled astrocytes and CD31 labeled endothelial cells (red), *n* = 5. (H,I): Immunofluorescence staining reveals the expression trends of LRP-1 in microglia, astrocytes, and endothelial cells in vitro injury environment. *n* = 5. *P < 0.05, **P < 0.01, ***P < 0.001.

The targeting action of Ang2 has been extensively studied in brain diseases, but the expression pattern of its receptor LRP-1 in the spinal cord and after SCI has not been clearly explored. To investigate the targeting specificity of Ang2 in the SCI area, we examined LRP-1 expression in various cell types following SCI. Data mining from online databases (GSE5296) revealed a significant increase in LRP-1 expression after SCI (Figure 1A). Subsequently, we extracted spinal cord tissues after injury for high-throughput sequencing, which confirmed the expression trend of LRP-1 (Figure 1B). The sequencing results showed a gradual rise in LRP-1 expression post-SCI, particularly between days 3–7, followed by a decline. Single-cell sequencing of spinal cord tissue (GSE189070) described the distribution of LRP-1 across different cell types, with high expression levels in microglia/macrophages and detectable levels in astrocytes and endothelial cells (Figure 1C,D). Consistent with transcriptome sequencing, LRP-1 expression significantly increased between days 3–7 post-SCI, then showed a declining trend by day 14 (Figure 1E).

We validated LRP-1 expression distribution in the spinal cord tissue of mice post-SCI using immunofluorescence experiments, which aligned with the single-cell sequencing results (Figure 1F,G). In vitro experiments observed LRP-1 expression levels; compared to controls, LRP-1 expression increased in microglia and astrocytes treated with LPS, and in endothelial cells treated with OGD/R (Figure 1H,I). These findings are consistent with previous studies.^27,28^ Our study confirmed that the expression trend of LRP-1 after SCI, providing guidance for the enrichment of Ang2 targeting peptide in the spinal cord. Thus, following SCI, the elevated expression of LRP-1 enhances the targeting specificity of Ang2 in the spinal cord, especially in microglia/macrophages.

### Construction and Characterization of Extracellular Vesicles from M2 Microglia

**Figure 2:**
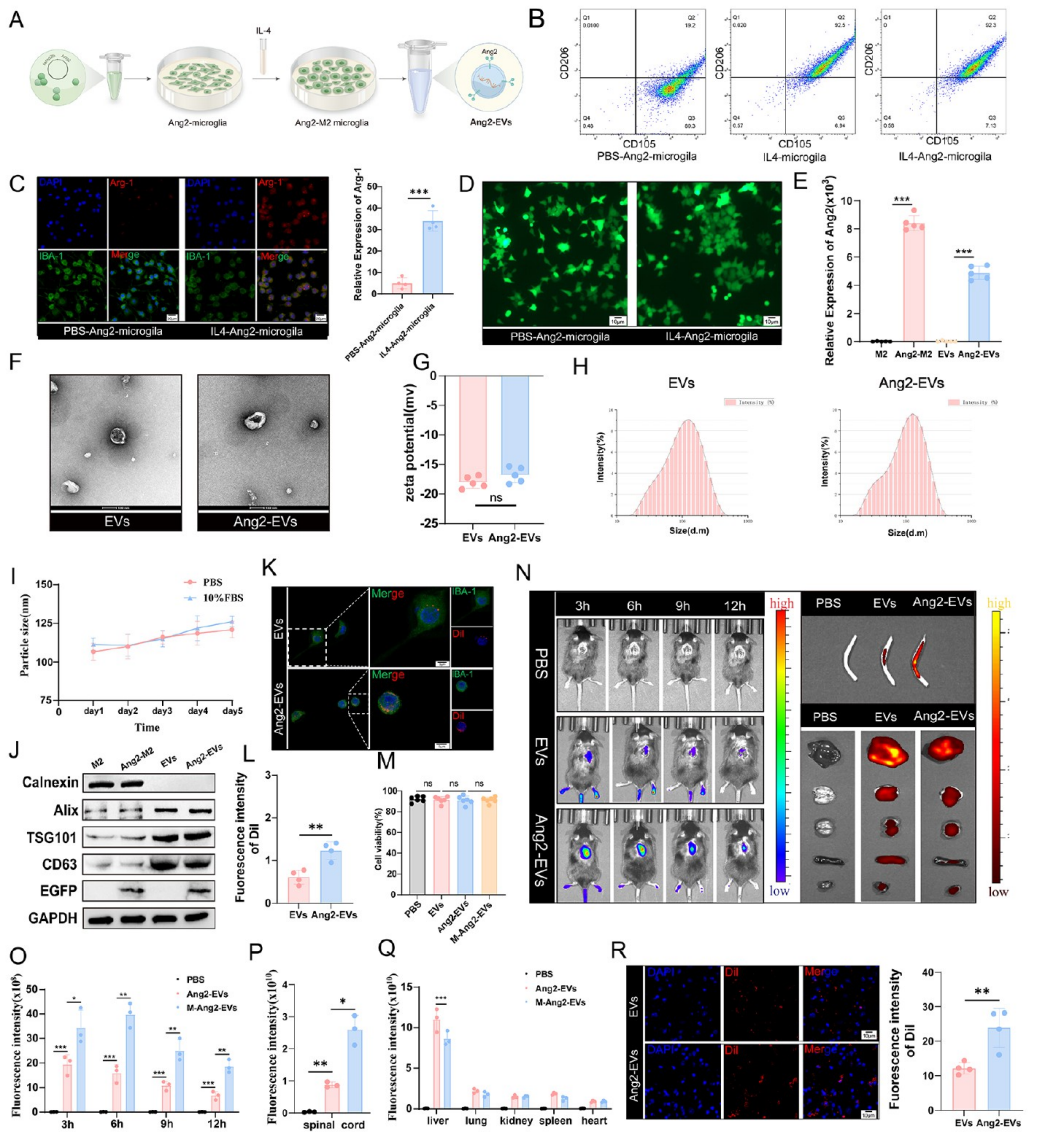
Preparation and characterization of Ang2-EVs. (A) The preparation process of extracellular vesicles derived from M2 microglia cells engineered with Ang2-targeting peptide. (B) Flow cytometry identifies the conversion efficiency of M2 microglia cells. (C) Immunofluorescence staining identifies the conversion efficiency of M2 microglia cells. Arg-1 labeled M2 microglia(red) and IBA-1 labeled microglia(green), *n* = 4. (D) EGFP green fluorescence represents successful integration of Ang2 into microglia cells. (E) qRT-PCR is used to identify the expression of Ang2 in M2 microglia cells and in extracellular vesicles derived from M2 microglia cells, validating the efficiency of Ang2 integration. *n* = 5. (F) TEM is used to observe the overall morphology of EVs and Ang2-EVs. (G) Zeta potential of EVs and Ang2-EVs. *n* = 5. (H) NTA analysis describes the diameter distribution of EVs and Ang2-EV. (I) The stability assessment of EVs and Ang2-EVs. *n* = 5. (J) Western blot analysis was used to identify surface markers of cells, EVs, and Ang2-EVs. (K,L) Observation of the targeting specificity and quantification of Dil-labeled Ang2-EVs in an in vitro simulated environment. *n* = 4. (M) CCK-8 assay detects the impact of Ang2-EVs on the viability of microglial cells. *n* = 6. (N) In vitro imaging shows the distribution of PBS, EVs, and Ang2-EVs in vivo, ex vivo spinal cord, and visceral organs. (O–Q) Quantitative distribution of PBS, EVs, and Ang2-EVs in vivo, spinal cord, and visceral organs. *n* = 3. (R) Fluorescence and quantitative analysis of the distribution of EVs and Ang2-EVs at the injury site in spinal cord tissue. *n* = 4. *P < 0.05, **P < 0.01, ***P < 0.001.

M2 microglia, known for their anti-inflammatory properties, were chosen as the source cells for EVs. Using the method of plasmid transfection, the Ang2 peptide labeled with EGFP was transfected into M0 microglia. Subsequently, IL-4 was used to differentiate M0 microglia into M2 microglia to obtain their secreted EVs (Figure 2A). Flow cytometry and immunofluorescence staining were employed to assess conversion efficiency, confirming that M2 microglia were CD11b-positive and CD206-positive, with elevated expression of the specific marker Arg-1 (Figure 2B,C). Culture medium collected from M2 microglia was subjected to ultracentrifugation to isolate EVs carrying the Ang2 peptide (Ang2-EVs), confirming transfection efficiency (Figure 2D). Real-time PCR analysis verified transcriptional expression levels of Ang-Lamp2b (Figure 2E). Western blot results demonstrated expression of Ang2-Lamp2b-EGFP in transfected M2 microglia and Ang2-EVs (Figure 2J). Electron microscopy revealed that Ang2-EVs maintained their spherical vesicle morphology (Figure 2F). As anticipated, Ang2-EVs exhibited a slightly larger particle size compared to EVs, while their zeta potential remained unchanged. Stability testing in PBS and 10% FBS showed consistent stability of Ang2-EVs over time in different solvents (Figure 2G–I). Western blot analysis identified surface markers of both cells and EVs (Figure 2J).

### Ang2 Peptide Enhanced the Targeting of EVs

To validate whether Ang2-EVs exhibit stronger targeting abilities compared to EVs, we conducted in vitro studies using BV2 cells expressing the highest levels of Ang2 receptors. EVs and Ang2-EVs were cultured with BV2 cells before and after LPS stimulation. We observed that LPS-stimulated BV2 cells enriched more Ang2-EVs (Figure 2K,L). Importantly, Ang2-EVs did not affect the viability of BV2 cells, confirming their safety (Figure 2M). Next, we investigated whether Ang2-EVs could accumulate at the injury site in vivo. Using IVIS imaging, we observed the distribution of Dil-labeled EVs and Ang2-EVs in mice. Results showed that both EVs and Ang2-EVs reached the spinal cord injury site, with Ang2-EVs demonstrating greater enrichment compared to unmodified EVs (Figure 2N,O). Fluorescence changes at the injury site over time postinjection revealed peak accumulation of Ang2-EVs at 6 h, followed by gradual metabolism. Ex vivo spinal cord imaging corroborated these trends observed in IVIS imaging (Figure 2N,P). We investigated the aggregation of Ang2-EVs in mouse organs to analyze the biological distribution of Ang2-EVs in vivo. The accumulation of Ang2-EVs in the liver was obvious, but less than that in the EVs group, and it was also expressed in other organs to different degrees (Figure 2N,Q). To confirm the presence of Ang2-EVs in spinal cord tissues, we performed immunofluorescence staining, revealing clustering of EVs and more pronounced clustering of Ang2-EVs around spinal cord tissue cells (Figure 2R). Thus, our engineered Ang2-EVs successfully targeted spinal cord injury sites, particularly in microglia/macrophages. MiRNAs are critical mediators of EVs’ biological functions. To elucidate Ang2-EVs’ biological roles, we sequenced their internal miRNA content and conducted GO and KEGG enrichment analysis on the top 10 miRNAs. Enrichment analysis highlighted their roles in biological processes, cellular components, and molecular functions, particularly in signaling pathways, membrane functions, protein binding, tumor pathways, and axon guidance (Figure S1A–C).

### Melatonin Pretreatment Enhances Ang2-EVs Functionality

**Figure 3:**
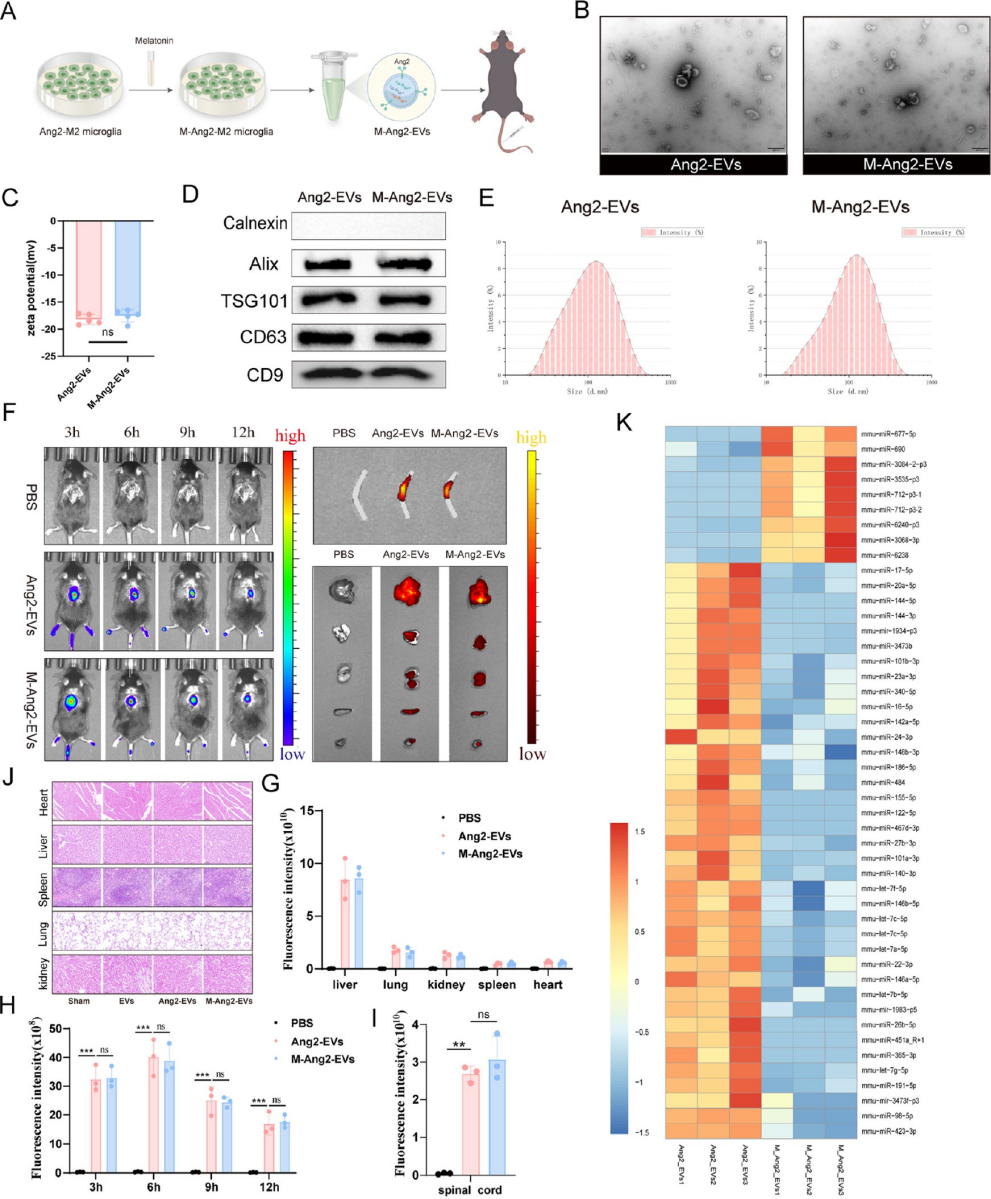
Construction and characterization of M-Ang2-EVs. (A) The preparation process of M-Ang2-EVs. (B) TEM comparison of the overall morphology of Ang2-EVs and M-Ang2-EVs. (C) Comparison analysis of the zeta potential between Ang2-EVs and M-Ang2-EVs. *n* = 5. (D) Western blot analysis to identify surface markers of Ang2-EVs and M-Ang2-EVs. (E) NTA for identifying the diameter distribution of Ang2-EVs and M-Ang2-EVs. (F) IVIS imaging shows the distribution of PBS, Ang2-EVs, and M-Ang2-EVs in vivo, ex vivo spinal cord, and visceral organs. (G–I) Quantitative analysis of PBS, Ang2-EVs, and M-Ang2-EVs in vivo, ex vivo spinal cord, and visceral organs. *n* = 3. (J) Representative HE staining of heart, liver, spleen, lung and kidney after treatment with PBS, EVs, Ang2-EVs and M-Ang2-EVs. (K) Heatmap depicting the differential miRNA expression between Ang2-EVs and M-Ang2-EVs. *P < 0.05, **P < 0.01, ***P < 0.001.

To enhance the biological function of EVs, we pretreated M2 microglia-derived EVs with melatonin, resulting in melatonin-pretreated M2 microglia-derived EVs (M-Ang2-EVs) (Figure 3A). As described previously, we characterized Ang2-EVs and M-Ang2-EVs separately (Figure 3B–E). The results indicated that the characteristics of Ang2-EVs did not significantly change after pretreatment. We compared the distribution of Ang2-EVs and M-Ang2-EVs in vivo, and extracellular imaging showed similar distribution and metabolic trends of Ang2-EVs and M-Ang2-EVs in the spinal cord and visceral organs, confirming that melatonin treatment does not affect the targeting ability of Ang2-EVs (Figure 3F–I). Subsequently, we evaluated the safety of engineered EVs after pretreatment in mice. Histological analysis of visceral morphology and blood parameters, including HE staining of heart, liver, spleen, lung, and kidney, showed intact organ structure without damage. Biochemical markers such as ALT confirmed that M2-EVs, Ang2-EVs, and M-Ang2-EVs did not exhibit toxicity in mice (Figures 3J and S2A). These findings demonstrate that M-Ang2-EVs engineered in this study have high biocompatibility and are nontoxic to the body.

We analyzed the miRNA expression profile of M-Ang2-EVs to investigate the biological functions conferred by melatonin treatment. Compared to untreated Ang2-EVs, M-Ang2-EVs showed 9 upregulated and 39 downregulated miRNAs (Figure 3K). Subsequently, we validated the upregulated and downregulated miRNAs and found that the expression levels of various miRNAs were generally consistent with the sequencing results, confirming the validity of our sequencing outcomes (Figure S6A). Functional enrichment analyses using GO and KEGG pathways were performed on the highly expressed miRNAs to predict their functions. GO analysis indicated that M-Ang2-EVs play roles in signal transduction, cellular membrane functions, and protein binding (Figure S3A–C). KEGG analysis revealed significant associations with pathways such as Endocytosis, Tight junctions, and Axonal guidance. These results elucidate the enhanced biological functionality of melatonin-pretreated Ang2-EVs (Figures 5A and 6A).

**Figure 4:**
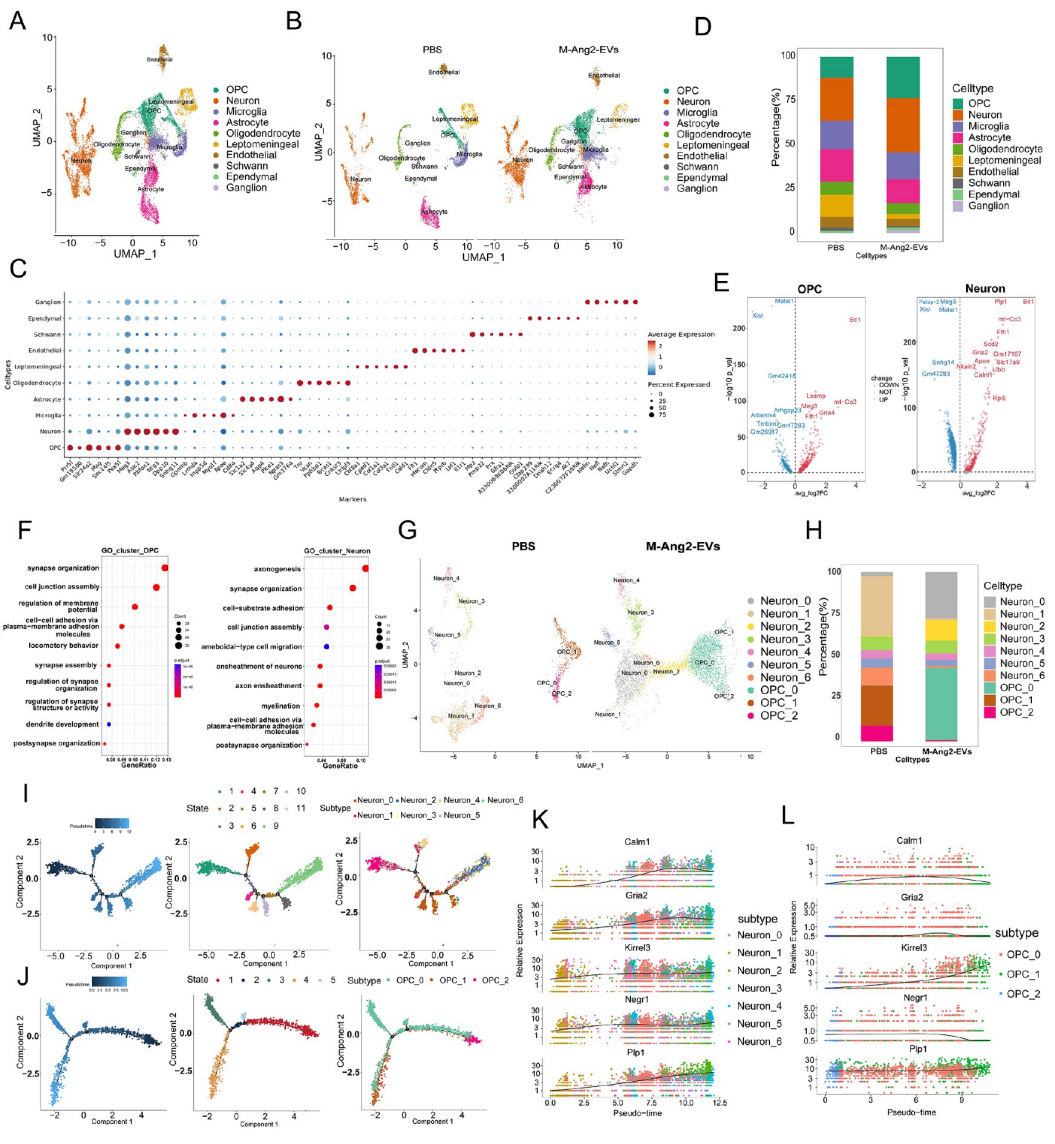
M-Ang2-EVs promote the reconstruction of nerve conduction function after spinal cord injury. (A,B) UMAP shows snRNA-seq analysis from PBS-and M-Ang2-EVs-treated mouse spinal cord cell populations. The color corresponds to the aggregation of cell populations. (C) Dot plot of the top six degs for each cluster. The color of the dot indicates the average RNA expression of the gene in the cell type, and the size of the dot indicates the percentage of cells in the cluster that express the gene. (D) Proportion of each cell population in the spinal cord of mice with SCI after treatment with PBS and M-Ang2-EVs. (E) Volcano plot of gene expression in OPC and Neuron populations in spinal cord tissue after treatment M-Ang2-EVs and PBS. (F) Results of GO enrichment analysis of genes highly expressed in OPC and Neuron populations in spinal cord tissue after M-Ang2-EVs treatment compared to PBS treatment. (G) UMAP visualization shows the distribution of neuron and OPC subsets. (H) Proportion of neuron and OPC subtype in the spinal cord of SCI mice treated with PBS and M-Ang2-EVs. (I) Pseudotime-ordered analysis of neuron subpopulation differentiation trajectories in spinal cord injury. (J) Pseudotime-ordered analysis of OPC subpopulation differentiation trajectories in spinal cord injury. (K,L) Dynamic plots show the expression of selected genes in neuron and OPC subsets.

**Figure 5:**
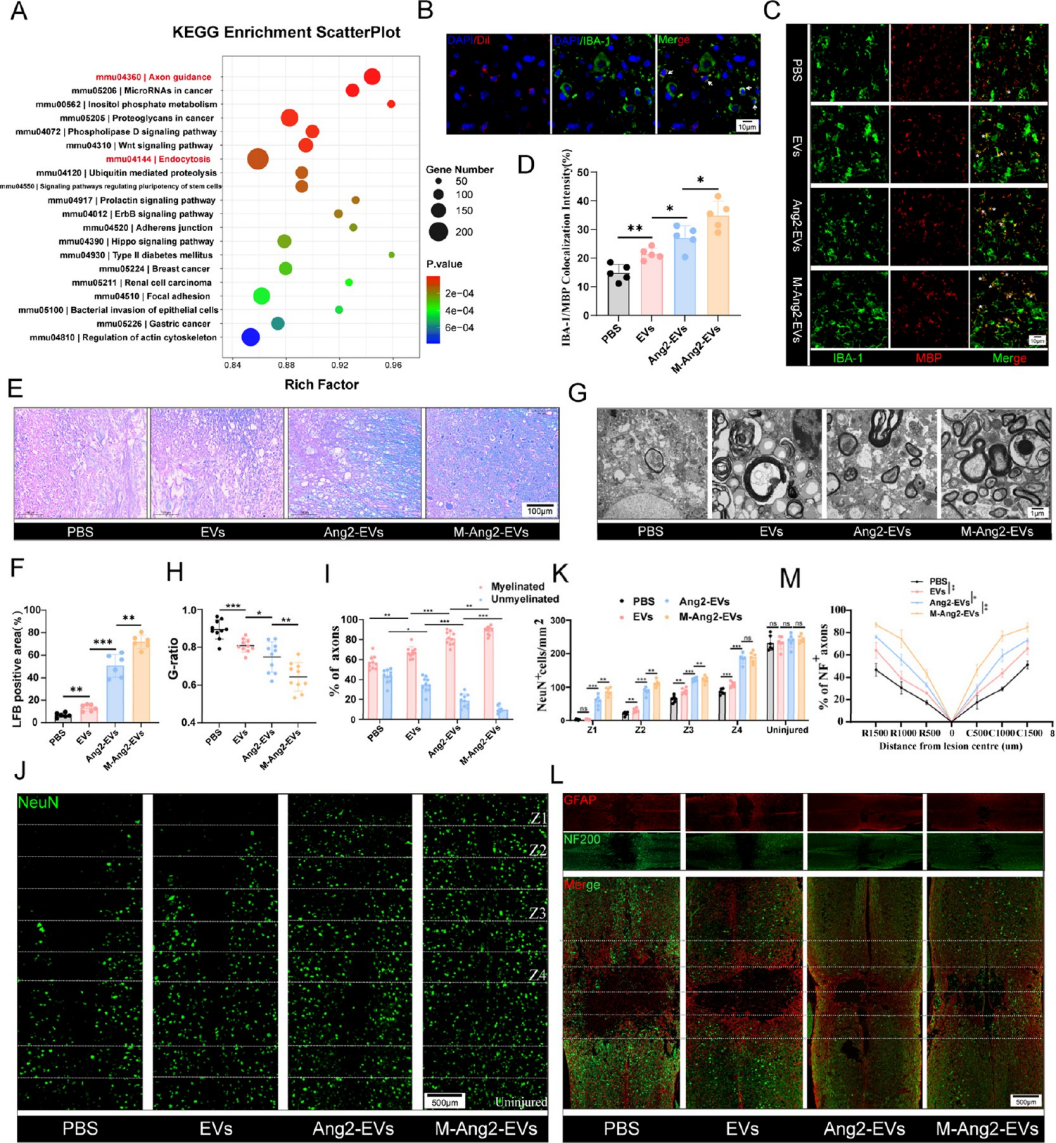
M-Ang2-EVs promote axonal remyelination and axon guidance by targeting microglia/macrophage. (A) The KEGG Enrichment ScatterPlot of differential genes between PBS and Ang2-EVs illustrates the biological functions of M-Ang2-EVs. (B) Dil-labeled M-Ang2-EVs are engulfed by microglia/macrophages at the injury site (Arrows show colocation). (C) Immunofluorescence staining demonstrates the phagocytosis of myelin debris by microglia/macrophages in different treatment groups, MBP labeled sphingomyelin (red), IBA-1 labeled microglia/macrophages (green) (Asterisk indicates colocation). (D) Quantification of myelin phagocytosis by microglia/macrophages at the spinal cord injury site after treatment with PBS, EVs, Ang2-EVs, and M-Ang2-EVs. *n* = 5. (E) LFB staining identifies the extent of axonal myelination at the spinal cord injury site after treatment with PBS, EVs, Ang2-EVs, and M-Ang2-EVs. (F) Quantification of LFB staining of axonal myelination at the spinal cord injury site after treatment with PBS, EVs, Ang2-EVs, and M-Ang2-EVs. *n* = 6. (G) TEM was used to observe the myelin morphology at the spinal cord injury site after treatment with PBS, EVs, Ang2-EVs, and M-Ang2-EVs. (H) G-ratio of axon myelin at the injury site after treatment with PBS, EVs, Ang2-EVs, and M-Ang2-EVs. *n* = 10. (I) Quantification of myelinated and unmyelinated axons at the injury site after treatment with PBS, EVs, Ang2-EVs, and M-Ang2-EVs. *n* = 10. (J,K): Representative immunofluorescence images and quantitative analysis of NeuN + neurons (green) in the Z1-Z4 regions near the core of the lesion at the injury site after treatment with different groups. *n* = 6. (L) Representative immunofluorescence images showing the distribution of astroglial scars and the growth of neuronal fibers at the injury site after treatment with different groups. GFAP labeled glial scars(red), NF200 labeled neurofilaments(green). (M) Quantification of NF200+ area in the center of spinal cord injury as a percentage of the total area of distal uninjured axons, *n* = 3. *P < 0.05, **P < 0.01, ***P < 0.001.

**Figure 6:**
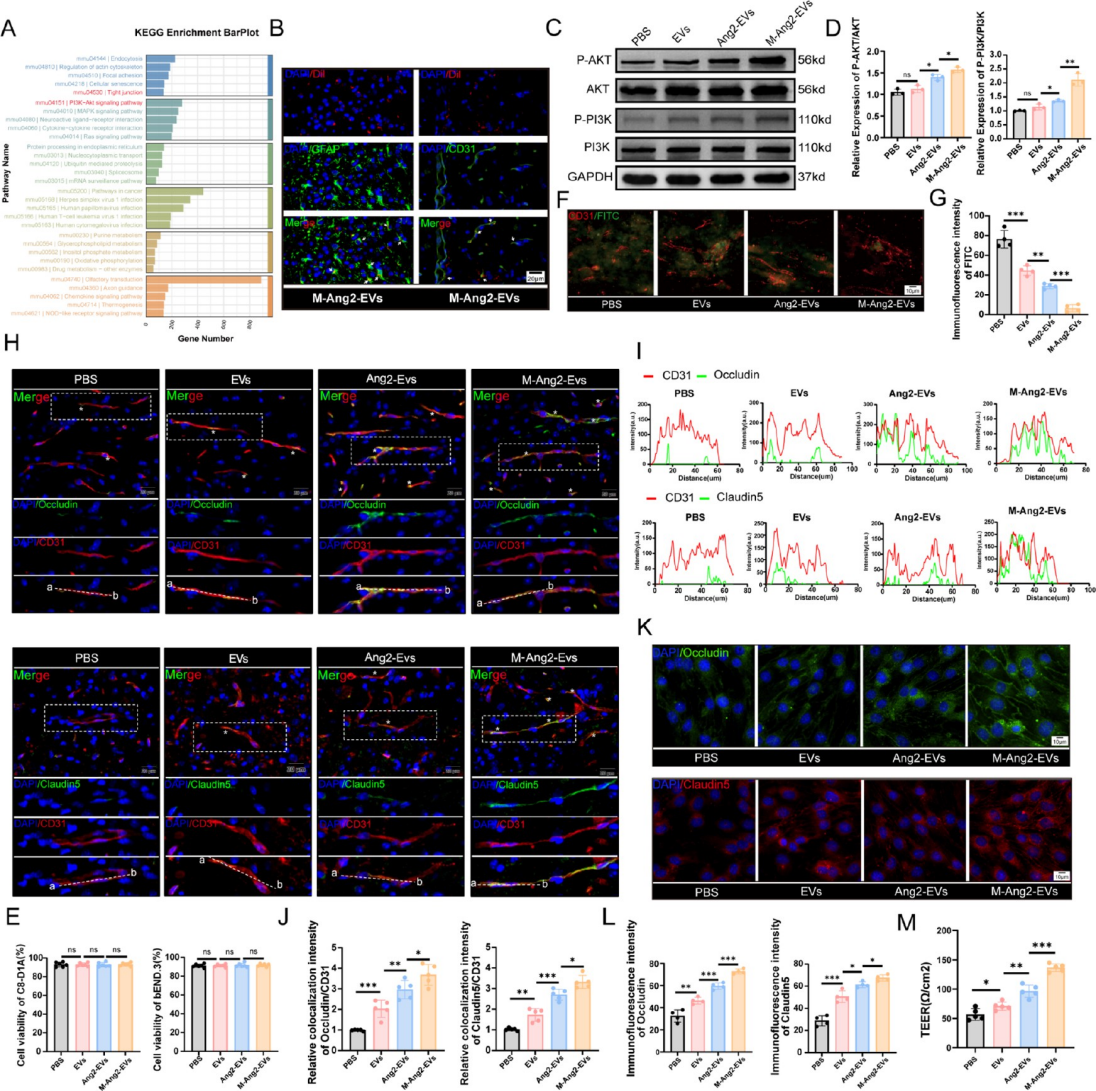
M-Ang2-EVs promotes blood-spinal barrier repair by targeting astrocytes and endothelial cells (A) The KEGG Enrichment BarPlot illustrates the biological functions of M-Ang2-EVs. (B) Representative immunofluorescence staining images showing M-Ang2-EVs engulfed by astrocytes and endothelial cells at the spinal cord injury site, (arrows show colocation). (C,D) Western Blot was used to detect the expression of PI3K-AKT pathway in spinal cord tissue under different treatments, *n* = 3. (E) CCK-8 evaluated the effects of PBS, EVs, Ang2-EVs, and M-Ang2-EVs on astrocyte and endothelial cell viability. *n* = 6. (F) FITC-Dextran evaluated the effects of PBS, EVs, Ang2-EVs, and M-Ang2-EVs on BSCB permeability at spinal cord injury sites. (G) Quantification of perivascular FITC-Dextran fluorescence intensity after PBS, EVs, Ang2-EVs, and M-Ang2-EVs treatment. *n* = 4. (H) Representative colocalization images of vascular tight junction proteins (Occludin, green) (Claudin5, green) with blood vessels (CD31, red) at the injury site following treatment with different groups, (Asterisk indicates colocation). (I) Line plot of colocalization intensity of Occludin and Claudin5 with CD31. (J) Quantitative analysis of the colocalization intensity of Occludin and Claudin5 with CD31. *n* = 5. (K,L) Immunofluorescence and quantization of Occludin and Claudin5 expression in bEnd.3 cells after PBS, EVs, Ang2-EVs, and M-Ang2-EVs treatment. *n* = 4. (M) Quantitative analysis of TEER in bEnd.3 cells after PBS, EVs, Ang2-EVs, and M-Ang2-EVs treatment. *n* = 5. *P < 0.05, **P < 0.01, ***P < 0.001.

### M-Ang2-EVs Promotes Axonal Regeneration and Nerve Conduction Recovery after Spinal Cord Injury

In order to elucidate the reparative and neuroprotective effects of M-Ang2-EVs on spinal cord injury, we collected spinal cord tissues from mice treated with PBS and M-Ang2-EVs at day 28 postinjury and performed single-cell nucleus sequencing. After excluding low-quality cells and potential doublets, we obtained 4992 and 7781 cells from the PBS-treated and M-Ang2-EVs-treated tissues, respectively. When visualized on a UMAP plot based on established marker gene expression and unsupervised cell type annotation, these cells clustered into ten different cell types, including OPC, neurons, microglia, astrocytes, oligodendrocytes, Leptomeningeal cells, oligodendrocytes, Endothelial cells, Schwann cells, Ependyma, Ganglion (Figure 4A,B). We identified the cell types by identifying the most significantly DEGs in each cluster (Figure 4C). Comparative analysis revealed that the proportion and gene expression levels of various cells in the spinal cord tissue had changed during the treatment process, especially with a significant increase in OPC crucial for myelination and neurons crucial for axon growth after M-Ang2-EVs treatment (Figure 4D). Differential analysis showed that compared to the PBS group, there were 274 upregulated genes and 250 downregulated genes in OPC cells following M-Ang2-EVs treatment. Neurons exhibited 326 upregulated genes and 817 downregulated genes (Figure 4E). GO enrichment analysis of upregulated genes after treatment indicated that M-Ang2-EVs play an important role in functions related to synapse organization, synapse assembly, regulation of synapse organization, regulation of synapse structure or activity for OPC; while neurons are involved in axonogenesis, synapse organization, axon ensheathment, myelination processes (Figure 4F). These results reveal the critical role of M-Ang2-EVs in axonal regeneration and repair following nerve injury. Furthermore, we also observed the functional and state changes of other cell types, and after M-Ang2-EVs treatment, the functional changes of most cells were in the direction of promoting spinal cord injury recovery (Figure S4).

As pivotal cells in the microenvironment after SCI, neurons exhibit multiple functions aimed at promoting axonal growth. To further investigate how M-Ang2-EVs impact neurons heterogeneity following spinal cord injury, samples from both PBS and M-Ang2-EVs treated group were further classified into seven transcriptionally distinct subpopulations (Neuron 0–6) for downstream analyses. OPC were also identified as distinct subpopulations (OPC 0–2), which helped delineate their roles within central nervous system development alongside functional recovery (Figures 4G and S5A,B). As shown in Figure 4H, the number of Neuron 1 and 6 was the highest in the PBS group, but it dramatically decreased after M-Ang2-EVs treatment, and the proportion of Neuron 0 as well as Neuron 2 began to increase. The number of other subtypes did not show significant changes. We hypothesize that Neuron 1 and 6 completed the transformation to Neuron 0 or Neuron 2 after the reasonable treatment. Similarly, the proportion of OPC 1 and 2 was significantly reduced after M-Ang2-EVs treatment compared to the PBS group, while the proportion of OPC 0 increased. In order to elucidate the developmental trajectories of neuron and OPC subsequent to the administration of therapeutically engineered extracellular vesicles, we performed pseudotime analysis on neuronal and OPC populations derived from both PBS and M-Ang2-EVs treated sample (Figure 4I,J). Consistent with our hypothesis, therapeutic intervention with M-Ang2-EVs induced the reprogramming of neuronal subgroups 1 and 6 into subgroups 0 and 2. The latter subgroups are characterized by their roles in modulating energy metabolism and facilitating synaptogenesis, which are essential for energy homeostasis and axonal outgrowth subsequent to spinal cord injury (Figure S5C). Additionally, we observed a gradual transition of OPC 2 toward the OPC 0 group, a shift that corresponds to the regulatory function of the OPC 0 group in the regulation of synapse organization (Figure S5D). The pseudotime order was utilized to illustrate the sequential fluctuations of genes associated with axonal neuroregeneration, thereby tracking the divergent cellular fates of distinct neuronal and OPC populations. The dynamic expression plot delineates an upregulation of Calm1, Gria2, Kirrel3, Negr1, and Plp1 along the pseudotime trajectory, transitioning from neuronal subgroups 1 and 6 to subgroups 0 and 2, before reaching a homeostatic plateau (Figure 4K). In the context of OPC, Calm1 and Negr1 exhibit a modest upward trend within the OPC 0 subset; however, the remaining genes do not display significant changes in expression patterns (Figure 4L).

Single-cell sequencing showed various cell function changes after M-Ang2-EVs treatment, especially in neural injury repair, from the extension of neuron axons and dendrites to the formation of myelin sheath and the release of neurotransmitters. The treatment of M-Ang2-EVs can enhance the nerve conduction function and play an important role in the reconstruction of nerve function after spinal cord injury.

### M-Ang2-EVs Promotes Microglia/Macrophages Phagocytosis and Axon Elongation

Previous studies have shown that microglia/macrophages phagocytosis of myelin debris promotes remyelination and axon growth, contributing to nerve injury repair.^29,30^ Our Single-cell sequencing and KEGG enrichment sequencing results indicated that M-Ang2-EVs are associated with cellular phagocytosis and axonal guidance (Figures 5A and S4). Microglia/macrophages are the main target cells of M-Ang2-EVs following spinal cord injury. Therefore, we conducted experiments to investigate whether M-Ang2-EVs promote microglia/macrophages phagocytosis of myelin debris and guide axon growth. Injection of M-Ang2-EVs into mice revealed that these vesicles were engulfed by microglia/macrophages cells in spinal cord tissue (Figure 5B). Subsequently, we examined the functionality of microglia/macrophages phagocytosis of myelin debris. Immunofluorescence staining showed that compared to PBS, EVs, and Ang2-EVs groups, IBA-1+ microglia/macrophages cells in the M-Ang2-EVs group exhibited more pronounced colocalization with MBP + myelin phospholipids, indicating enhanced clearance of myelin debris post M-Ang2-EVs treatment (Figure 5C,D). Effective clearance of myelin debris promotes axonal remyelination. We observed levels of remyelination using LFB staining post injury, where mice treated with M-Ang2-EVs demonstrated robust remyelination capability compared to limited efficacy with EVs and poorest with PBS treatment (Figure 5E,F). TEM evaluation of myelin integrity revealed extensive demyelination in the PBS group, with vesicular and fragmented myelin even after EVs and Ang2-EVs treatments, while the M-Ang2-EVs group showed well-preserved spinal cord myelin sheaths with distinct multilayered structures of myelin phospholipids (Figure 5G). Quantification of the G ratio and counts of myelinated and demyelinated axons showed a significantly higher axon diameter to outer diameter ratio in the PBS group compared to other treatment groups, with the smallest ratio observed in the M-Ang2-EVs group. Furthermore, the number of demyelinated axons decreased while myelinated axons increased progressively from PBS to EVs to Ang2-EVs and finally to M-Ang2-EVs groups, indicating effective myelin preservation (Figure 5H,I).

Neuronal survival is crucial for restoring motor function in mice. Using NeuN labeling to mark neurons and categorizing regions from Z1 near the injury to Z4 further away, M-Ang2-EVs treatment consistently showed higher neuronal survival, suggesting M-Ang2-EVs can mitigate neuronal death (Figure 5J,K). NF200 labeled axons in the spinal cord to observe their growth status, while GFAP marked glial scars in the injury area. In the PBS group, neuronal fiber extension was obstructed post-treatment, whereas EVs treatment showed some increase in neuronal fiber length. However, after M-Ang2-EVs treatment, neuronal fibers significantly extended across the scar in the injury area, correlating with the enhanced remyelination and axonal guidance functions of M-Ang2-EVs (Figure 5L,M).

### M-Ang2-EVs Promote Blood-Spinal Cord Barrier Repair by Enhancing Tight Junction Protein Expression

The disruption of the BSCB following SCI is a critical factor in the development of secondary inflammation. Astrocytes and endothelial cells are essential components of the BSCB.^31^ Our previous results indicated that, in addition to targeting microglia, M-Ang2-EVs partially target astrocytes and endothelial cells. The sequencing results also highlighted the regulatory role of M-Ang2-EVs in the PI3K-Akt signaling pathway and vascular tight junctions, and the expression of PI3K-Akt signaling pathway in spinal cord tissues was verified by Western Blot (Figure 6A,C,D). It is reasonable to assume that after being phagocytosed by astrocytes and endothelial cells, M-Ang2-EVs can promote vascular tight junction repair. We observed the phagocytosis of M-Ang2-EVs by astrocytes and endothelial cells (Figure 6B) and confirmed that this did not affect the viability of these cells (Figure 6E). We compared the phagocytosis of M-Ang2-EVs by other resident cells within the spinal cord tissue (oligodendrocytes, neurons). Although these cells also have some phagocytic activity, their relatively low phagocytosis rates demonstrate the targeting effect of Ang2 (Figure S6B). Next, we injected FITC-Dextran into mice to observe its penetration in spinal cord tissue. Quantification of spontaneous fluorescence of FITC-Dextran revealed that M-Ang2-EVs significantly reduced FITC-Dextran diffusion around blood vessels in the injury area (Figure 6F,G). We then examined changes in tight junctions (TJs) proteins, Occludin and Claudin5, at the SCI site following M-Ang2-EVs treatment. Immunofluorescence showed a significant reduction in Occludin protein levels and poor colocalization with CD31-labeled blood vessels post-SCI. Treatment with EVs, Ang2-EVs, and M-Ang2-EVs progressively increased Occludin expression and enhanced its colocalization with CD31 (Figure 6H,J). Claudin5 expression exhibited a similar trend (Figure 6H,J). In an in vitro OGD/R model using bEnd.3 cells to simulate SCI conditions, we observed that TJs protein expression was higher in the treatment groups compared to the PBS group (Figure 6K,L), indicating that M-Ang2-EVs promoted endothelial cell tight junction recovery in vitro. Finally, transepithelial electrical resistance was measured in different treatment groups to determine the integrity of endothelial tight junctions after M-Ang2-EVs treated (Figure 6M). Consistent with the immunofluorescence and in vivo results, EVs could promote the recovery of TJs, with M-Ang2-EVs being the most effective. Both in vivo and in vitro experiments confirm that M-Ang2-EVs facilitate BSCB repair after SCI.

### M-Ang2-EV Alleviates Secondary Inflammatory Responses Following Spinal Cord Injury

We have established the protective role of M-Ang2-EV on the BSCB, which mitigates vascular permeability to inflammatory factors, safeguards the spinal cord parenchyma from secondary inflammatory damage, and modulates the immune microenvironment homeostasis at the injury site. Additionally, M-Ang2-EV demonstrates targeting capability toward microglia/macrophages, polarization of which plays a pivotal role in regulating secondary inflammatory responses. Consequently, we investigated the impact of M-Ang2-EV on oxidative stress and inflammatory cytokines at the injury site to confirm its modulatory effects on the spinal cord microenvironment.

**Figure 7:**
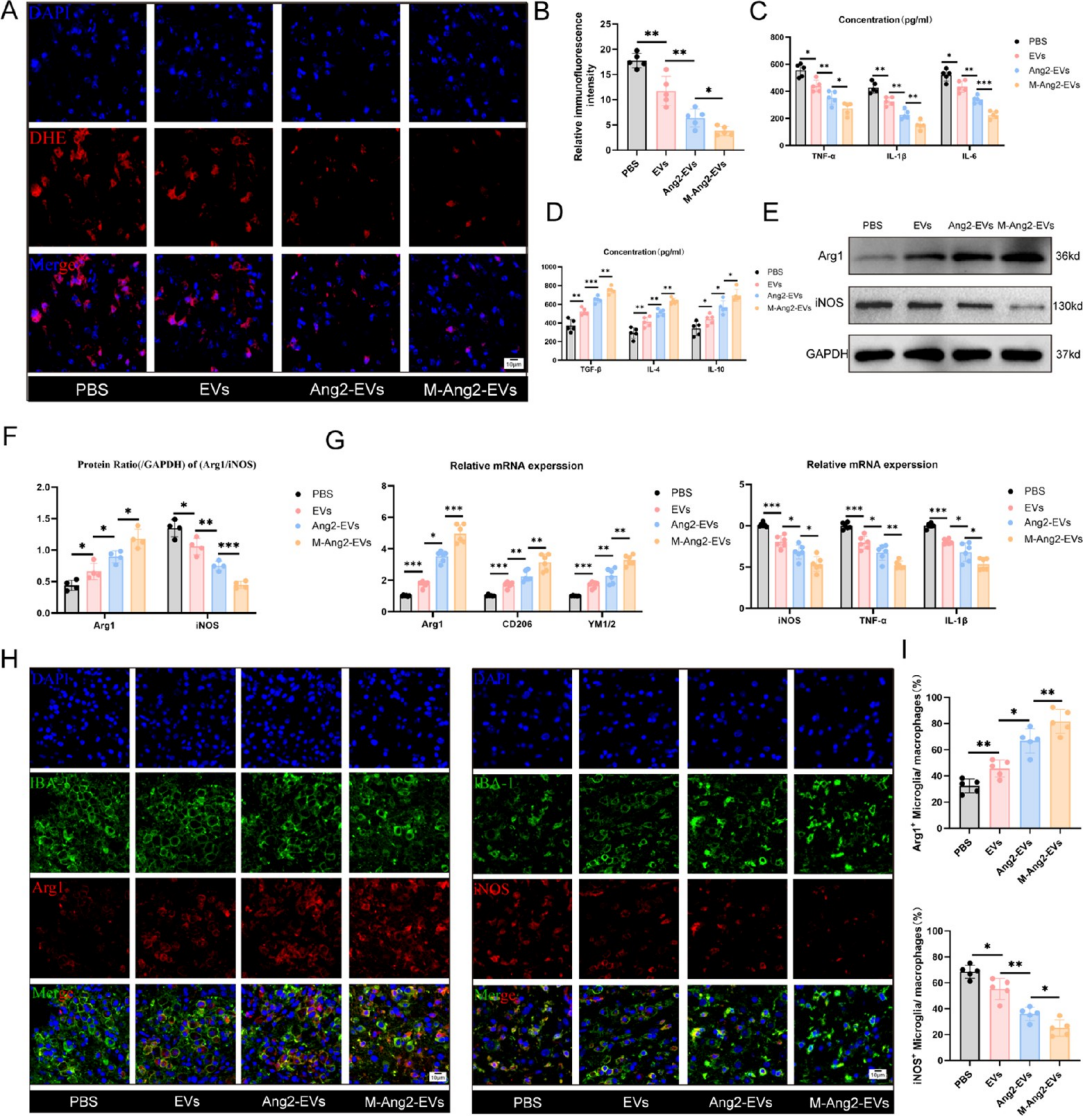
M-Ang2-EVs prevents inflammation progression after SCI. (A) DHE staining elucidates the degree of oxidative stress at the site of spinal cord injury following treatment with various therapeutic groups (DHE: red). (B) Quantitative analysis of DHE immunofluorescence staining. *n* = 5. (C) ELISA assesses the expression of proinflammatory cytokines (TNF-α, IL-1β, IL-6) at the site of spinal cord injury after treatment with different therapeutic groups. *n* = 5. (D) ELISA evaluates the expression of anti-inflammatory cytokines (TGF-β, IL-4, IL-10) at the site of spinal cord injury post-treatment with various therapeutic interventions. *n* = 5. (E,F) Western Blot analysis and quantitative assessment of the expression of markers for different subtypes of microglia/macrophages (M1: iNOS, M2: Arg1) at the site of spinal cord injury after treatment with PBS, EVs, Ang2-EVs, and M-Ang2-EVs. *n* = 4. (G) qRT-PCR examination of the expression of markers for different subtypes of microglia/macrophages (M1: iNOS, TNF-α, IL-1β; M2: Arg1, CD206, YM1/2) at the site of spinal cord injury following treatment with PBS, EVs, Ang2-EVs, and M-Ang2-EVs. *n* = 6. (H,I) Immunofluorescence staining for markers of M1/M2 subtypes of microglia/macrophages (IBA-1) and quantitative analysis of the number of positive cells. *P < 0.05, **P < 0.01, ***P < 0.001.

Initially, we assessed the level of oxidative stress postspinal cord injury. DHE staining revealed a significant increase in reactive oxygen species (ROS) following injury. Treatments with EVs, Ang2-EVs, and M-Ang2-EVs progressively reduced oxidative stress levels, with M-Ang2-EVs exhibiting the most pronounced effect (Figure 7A,B). Subsequently, ELISA was employed to detect the concentration trends of pro-inflammatory cytokines TNF-α, IL-1β, and IL-6 in the spinal cord. Compared to EVs and Ang2-EVs, M-Ang2-EVs significantly decreased the concentrations of these pro-inflammatory cytokines. We also analyzed the concentrations of anti-inflammatory cytokines TGF-β, IL-4, and IL-10, which exhibited opposite trends compared to the pro-inflammatory cytokines (Figure 7C,D). To observe the polarization status of microglia/macrophages, Western blot analysis was used to assess the expression of M1 phenotype markers (iNOS) associated with pro-inflammatory activity and M2 phenotype markers (Arg1) associated with anti-inflammatory activity. The results showed that M-Ang2-EVs significantly increased the expression of M2 microglia/macrophage markers while decreasing the expression of M1 markers (Figure 7E,F). qRT-PCR analysis across different groups showed a similar trend (M1: iNOS, TNF-α, IL-1β; M2: Arg1, CD206, YM1/2) (Figure 7G). Further observation of microglia/macrophage phenotypic transformation within spinal cord tissue was conducted using immunofluorescence staining. The PBS-treated group showed low Arg1 expression and high iNOS expression, but after treatment with the three EVs, Arg1 expression gradually increased while iNOS expression decreased, confirming the ability of M-Ang2-EVs to promote the transition of M1 microglia/macrophages to M2 and alleviate inflammation at the injury site (Figure 7H,I). These findings suggest that M-Ang2-EVs can alleviate secondary inflammation following spinal cord injury, regulate the microenvironment at the injury site, and promote injury repair.

### M-Ang2-EVs Alleviate Brain Inflammation Following Spinal Cord Injury

**Figure 8:**
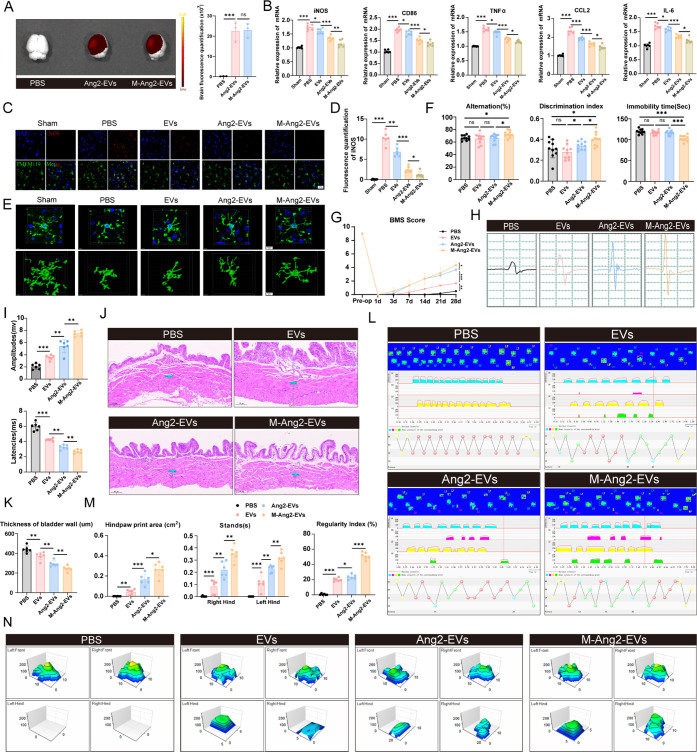
M-Ang2-EVs can promote the recovery of cognitive function and motor function after spinal cord injury (A) IVIS imaging and quantification shows accumulation of Ang2-EVs and M-Ang2-EVs in the brain. *n* = 3. (B) Quantification of brain inflammation-related markers following different treatment modalities in Sham and SCI conditions. *n* = 6. (C,D) Representative immunofluorescence staining and quantitative analysis of the M1 polarization marker iNOS in microglia in the brain following different treatment modalities in Sham and SCI conditions, iNOS labeled M1 polarized microglia marker(red), TEME119 labeled microglia(green). *n* = 6. (E) Three-dimensional reconstruction of microglial morphology in the brain following different treatment modalities in Sham and SCI conditions. (F) Quantification of Y-maze, NOR test, and TS results to assess the improvement in spatial cognitive ability and depressive behavior in SCI mice. *n* = 10. (G) BMS score assesses the motor function of spinal cord injury mice after treatment with PBS, EVs, Ang2-EVs, and M-Ang2-EVs. (H) Electrophysiological assessment with MEP analysis of SCI mice after treatment with PBS, EVs, Ang2-EVs, and M-Ang2-EVs. (I) Quantification of peak-to-peak MEP amplitude and latency of SCI mice after treatment with PBS, EVs, Ang2-EVs, and M-Ang2-EVs. *n* = 6. (J) Bladder wall thickness was used to evaluate bladder recovery in SCI mice after treatment. (K) Quantification of bladder wall thickness. *n* = 6. (L,N) Representative paw prints, hind paw pressure images and temporal views of limb support from Catwalk gait analysis. (M) Quantitative analysis of regularity index, stands, and hind paw print area in Catwalk gait analysis of spinal cord injury mice following different treatment modalities. *n* = 6. *P < 0.05, **P < 0.01, ***P < 0.001.

Due to the distribution of LRP-1 in the mouse brain, Ang2 peptide-modified EVs can partially target brain tissue (Figure 8A). Studies have shown that SCI induces brain inflammation, which hinders spinal cord repair and leads to cognitive impairment and depressive behaviors.^32^ Chronic inflammation mainly occurs in the cerebral cortex, hippocampus and hindbrain. Taking the cerebral cortex as an example, we investigated whether M-Ang2-EVs could alleviate brain inflammation. Following SCI, microglia in the cerebral cortex polarize toward the M1 phenotype. We assessed the RNA expression of M1 microglia markers and found that the presence of EVs reduced the expression of iNOS, CD86, TNFα, CCL2, and IL-6, with M-Ang2-EVs showing the most significant reduction (Figure 8B). Similarly, EVs and Ang2-EVs decreased iNOS expression in M1 microglia in the brain, with M-Ang2-EVs being particularly effective (Figure 8C,D). Using laser confocal microscopy, we observed the morphology of brain microglia after SCI and performed three-dimensional reconstruction. In the Sham group, microglia exhibited distinct branching, while in the SCI PBS-treated group, the microglia became rounded with fewer processes. Treatment with M-Ang2-EVs preserved microglial morphology, indicating that these extracellular vesicles can prevent morphological changes in microglia (Figure 8E). Based on these results, we conclude that targeted extracellular vesicles, such as M-Ang2-EVs, can significantly reduce brain inflammation following spinal cord injury. Therefore, we further investigated the effects of M-Ang2-EVs on brain function. Spatial memory and depressive-like behavior were assessed in mice 10 weeks postinjury using the Y-maze, NOR test, and Tail Suspension Test. While regular EVs treatment did not affect spatial memory and depressive-like behavior, M-Ang2-EVs treatment significantly increased alternation and discrimination index and decreased immobility time, suggesting improvements in spatial memory and reduction in depressive-like behavior (Figure 8F).

### M-Ang2-EVs Promoted Motor Function Recovery and Improved Cognitive Impairment and Depression in Mice

Our research has demonstrated the role of Ang2-EVs and M-Ang2-EVs in promoting spinal cord injury recovery in mice through various biological processes. We further investigated their effects on motor function. The BMS score, commonly used to assess balance and hind limb recovery in mice, showed that EVs treatment resulted in better motor function compared to the PBS group. The group treated with targeted peptides, particularly with melatonin pretreatment, showed even more significant improvements (Figure 8G). Electrophysiology assessments of hind limb nerve conduction in spinal cord injured mice revealed that the mice treated with M-Ang2-EVs had significantly larger motor evoked potential (MEP) amplitudes and shorter latencies compared to the PBS group, indicating improved neural conduction (Figure 8H,I). Bladder wall thickness, an indicator of bladder function recovery,^33^ was also reduced in the M-Ang2-EVs group compared to the PBS group, which exhibited thickened bladder walls and increased collagen content (Figure 8J,K). The Cat Walk system was utilized to evaluate motor function recovery in mice by analyzing gait coordination, hind limb support points, and other related indicators. We observed that SCI mice treated with PBS were unable to support their hind paws on the ground. However, after treatment, the hind limb movement rhythm gradually became regular, particularly in the M-Ang2-EVs group (Figure 8L,N). Indicators such as regularity index, stands, and hind paw print area significantly improved after treatment, indicating that M-Ang2-EVs contribute to better motor function recovery in mice (Figure 8M). These results indicate that M-Ang2-EVs provide dual therapeutic benefits, enhancing both spinal cord and brain function, thereby improving spatial memory and motor function in mice.

## Discussion

Spinal cord injury is a severe condition primarily caused by car accidents, violence, and falls from heights, resulting in high mortality and disability rates, and imposing a significant burden on patients’ families.^34,35^ The sudden onset and complex pathophysiology of SCI make its treatment particularly challenging, prompting researchers to seek effective therapeutic approaches continuously.^36^

Extracellular vesicles, small vesicles formed by cell membrane budding, have gained increasing importance in SCI research and treatment, the choice of donor cells for EVs is crucial for their function.^37^ Previous studies have demonstrated that EVs derived from bone marrow mesenchymal stem cells, adipose mesenchymal stem cells, umbilical cord mesenchymal stem cells, and neural stem cells have significant therapeutic potential in SCI treatment.^38−40^ In this study, we selected M2 microglia as the source of EVs due to their proven anti-inflammatory properties and ease of acquisition, given the persistent inflammatory cascade following SCI. EVs can evade immune rejection and cross the blood-spinal barrier, making it feasible for them to reach and act on SCI lesions. However, regardless of whether they are administered via tail vein injection or local delivery, a significant amount of EVs is inevitably lost, limiting the number of EVs that can exert therapeutic effects and thereby restricting their overall efficacy. To address this issue, we modified the EVs to enhance their targeting to the injury site. Additionally, we employed melatonin preconditioning to augment the functionality of individual EVs, thereby improving the overall therapeutic efficiency for SCI.

The oligopeptide Angiopep-2, known for its high transcytosis capacity across the blood–brain barrier (BBB), has been used as a targeting ligand in combination with nanoparticles or EVs for targeted delivery.^41^ Ang2 peptides were initially employed to treat brain diseases, including cancer, brain injury, stroke, epilepsy, fungal infections, Alzheimer’s disease (AD), and Parkinson’s disease (PD).^20,42,43^ However, their application in spinal cord diseases remains unexplored. For Ang2 peptides to exert their targeting function, a crucial requirement is the presence of LRP-1 receptors, which are highly expressed in the BBB. To explore this, we analyzed the transcriptome sequencing data of LRP-1 in the spinal cord. Excitingly, transcriptome results indicated that LRP-1 is expressed in the spinal cord and its expression is elevated from day 3 to day 7 post-SCI. This pattern mirrors the infiltration trends of microglia/macrophages, suggesting a potential correlation. Therefore, we conducted single-cell sequencing at various time points post-SCI to observe cell population distribution and quantify gene expression within these populations. We found that LRP-1 is highly expressed in microglia/macrophages post-SCI and is also present in astrocytes and endothelial cells, aligning with our hypothesis. This indicates that Ang2 peptides have a specific receptor cell population in the injured spinal cord. On this basis, we used plasmids to transfect the Ang2 peptide and lamp2b into M0 microglia. Lamp2b anchors Ang2 to the cell membrane, ensuring that vesicles budding from the membrane also carry Ang2 peptides. Subsequently, we used IL-4 to convert M0 microglia into anti-inflammatory M2 microglia. When EVs and Ang2-EVs were injected into mice, in vivo imaging revealed significantly higher distribution of Ang2-EVs in the spinal cord and slightly reduced distribution in visceral organs, confirming the targeted action of Ang2 on the injured spinal cord.

Through these steps, we successfully increased the quantity of EVs enriched at the injury site. To further enhance the quality of EVs, we explored various strategies. These modifications significantly improved the efficacy of SCI treatment by increasing the functional capabilities of individual EVs.

Melatonin is a pineal hormone associated with circadian rhythms, regulating physiological processes and maintaining homeostasis. It is synthesized by various tissues and organs, including the heart, liver, placenta, skin, kidneys, and intestines.^25^ Increasing evidence indicates that melatonin exhibits anti-inflammatory effects in both acute and chronic inflammatory processes. Studies have shown that exogenous melatonin can enhance serum levels of the anti-inflammatory cytokine IL-4 and reduce levels of pro-inflammatory cytokines such as interleukin-1β (IL-1β) and tumor necrosis factor-α (TNF-α) in rats.^44^ Our previous research also demonstrated that melatonin preconditioning of mesenchymal stem cell-derived EVs stabilized NRF2 through ubiquitination processes, aiding in the repair of traumatic spinal cord injury, thereby confirming melatonin’s role in spinal cord injury.^45^ Based on these findings, we pretreated anti-inflammatory microglia with melatonin. miRNAs, a class of small endogenous noncoding RNAs, play a crucial role in the functionality of EVs.^46,47^ By binding to complementary target mRNAs, miRNAs inhibit mRNA translation, thereby negatively regulating gene expression at the post-transcriptional level.^48^ This mechanism is pivotal for the therapeutic effects of EVs in inflammatory and injury contexts. MiRNAs are involved in almost all developmental and pathophysiological processes, including various forms of cell death, differentiation, proliferation, inflammation, and immune responses.^49^ They play a crucial role in the pathogenesis of inflammatory diseases, including spinal cord injury. We sequenced the miRNAs in melatonin-treated M-Ang2-EVs and performed enrichment analysis on the highly expressed miRNAs. We focused on the endocytosis function because the targeted microglia by M-Ang2-EVs have strong phagocytic capabilities, which are closely related to the repair of SCI.

Next, in order to determine the effect of M-Ang2-EVs on neural function in mice with spinal cord injury, single nucleus sequencing was performed on spinal cord tissue at the injury site on day 28 after spinal cord injury. OPC and neuronal cells increased significantly after M-Ang2-EVs treatment. Since OPC cells are the main cells involved in myelination, the treatment of engineered extracellular vesicles can ensure the survival of neurons at the injured site and the remyelination of axons, which perfectly matches the results of our M-Ang2-EVs miRNAs sequencing and immunofluorescence staining above. As anticipated, the treatment with M-Ang2-EVs led to a significant increase in cell subtypes (Neuron0 and 2, OPC 0), are known to modulate energy homeostasis and facilitate the reconstruction of neural circuitry. This finding elucidates the role of M-Ang2-EVs in facilitating the repair of spinal cord injuries and the recovery of motor function. Increasing evidence suggests that the persistent presence of myelin debris in damaged spinal cords not only inhibits axon regeneration but also acts as a potent inflammatory stimulus, promoting demyelination processes and potentially exacerbating tissue damage.^50^ Therefore, the clearance of myelin debris is critical for SCI repair. Additionally, microglia/macrophages can clear myelin debris, helping to suppress neuroinflammation and promote axon regeneration.^30,51,52^ Interestingly, melatonin-treated Ang2-EVs can enhance the phagocytic function of microglia/macrophages, single cell sequencing also revealed that M-Ang2-EVs was associated with axonal regeneration. We confirmed this enhancement using immunofluorescence staining. By leveraging melatonin’s preconditioning effects on microglia, we aim to optimize the therapeutic potential of M-Ang2-EVs in promoting effective debris clearance and facilitating the repair processes in SCI. On this basis, we evaluated post-treatment myelination using LFB staining and electron microscopy. We found that M-Ang2-EVs treatment effectively promoted axonal remyelination. Inflammation and other changes in the local environment after SCI can alter the expression of axon guidance systems. After SCI, ascending and descending axonal tracts are damaged, and longitudinal connections are difficult to repair. Intense secondary injury makes it challenging for axons to penetrate scar tissue and grow long distances, especially in the injured area.^53^ Axon guidance mechanisms, which regulate the direction and path of axonal growth, play a critical role after injury.^54^ Considering the enrichment of axon guidance KEGG terms in the gene characteristics of M-Ang2-EVs, we examined neuronal survival and axonal growth. We performed neuronal partitioning and localization, finding that a significant number of NeuN + neurons survived at the injury site post-treatment. In contrast, many neurons die following SCI. Additionally, targeted EVs treatment reduced astrocytic scarring at the injury site, and neurofilament extension was evident, even reaching the injury center. This observation aligns with the axon guidance function of M-Ang2-EVs. Therefore, the enhanced targeting capability and the use of melatonin significantly promoted axonal growth and myelin regeneration, contributing positively to the restoration of neural conduction functions postinjury.

The BSCB is a barrier within the spinal cord that prevents toxins, blood cells, and pathogens from entering, maintaining strict chemical balance in the spinal cord environment.^31^ It plays a crucial protective and regulatory role in spinal cord parenchyma. Functionally similar to the BBB, the BSCB consists of endothelial cells, intercellular junctions, pericytes, basement membrane, and astrocytic end-feet, among other structures. These complex components determine the protective and regulatory functions of the BSCB.^55,56^ M-Ang2-EVs can target astrocytes and endothelial cells in the injured spinal cord, potentially facilitating repair of the BSCB after SCI. According to KEGG terms, M-Ang2-EVs regulate the PI3K-Akt signaling pathway and tight junction. The PI3K-Akt signaling pathway has been shown to promote BSCB repair,^57^ and tight junctions are critical for BSCB integrity. Enhanced expression of TJ proteins can maintain BSCB integrity. Thus, we conducted colocalization analysis of TJ protein expression and vascular markers in the injured spinal cord after treatment with EVs, Ang2-EVs, and M-Ang2-EVs. We found that EVs, Ang2-EVs, and M-Ang2-EVs all increased the expression of Occludin and Claudin-5, enhancing their colocalization with CD31. Notably, M-Ang2-EVs exhibited more pronounced effects, highlighting the significant role of melatonin. Building on this, we observed reduced permeability of FITC-Dextran after treatment with M-Ang2-EVs, indicating its enhanced ability to maintain BSCB integrity. The integrity of the BSCB serves as a barrier against the infiltration of inflammatory factors, thereby safeguarding the spinal cord parenchyma. Our findings reveal that the presence of M-Ang2-EVs protects the immune microenvironment of the spinal cord and alleviates secondary inflammation. This protective effect may be attributed to the indirect action of M-Ang2-EVs on the spinal cord parenchyma through their preservation of BSCB integrity, as well as the direct modulation of microglia/macrophages by M-Ang2-EVs.

Severe spinal cord injury can lead to neurobehavioral abnormalities and neuropathophysiological changes that cause cognitive impairments, including deficits in learning, memory, executive function, attention, and processing speed.^58^ It often results in depression, impacting the psychological health of patients. Research has shown that after spinal cord injury, there is organic damage in the brain, particularly in the hippocampus and cerebral cortex. Microglial cells tend to polarize toward the M1 phenotype, accompanied by increased inflammatory factors, which adversely affect brain health and hinder the repair process of spinal cord injury.^32,59^ Therefore, mitigating brain inflammation after spinal cord injury is as crucial as treating the spinal cord injury itself, and reducing brain inflammation may also promote spinal cord function recovery. Ang2 peptide can target the blood–brain barrier. Therefore, after programming targeting peptides into EVs, besides targeting the spinal cord injury site, a portion of Ang2-EVs and M-Ang2-EVs inevitably target the brain, presenting an opportunity to treat brain inflammation. We confirmed this hypothesis through experiments, observing that targeted treatment slowed down microglial cell polarization, preserving their branching and reducing markers associated with M1 polarization. Building on this, cognitive impairments and depression induced by long-term spinal cord injury in mice were alleviated to some extent. We believe that M-Ang2-EVs can exert protective effects on the brain in two ways: one is by directly targeting the brain to exert anti-inflammatory effects, and the other is by indirectly suppressing brain inflammation by promoting the recovery of spinal cord injury. However, it is not yet known which method plays the primary role. Given that chronic inflammation may be widespread in multiple regions of the brain, and we only studied the inflammatory response in the cerebral cortex, whether M-Ang2-EVs can improve inflammation throughout the entire brain is a blind spot in our research. However, LRP-1 can be expressed in the blood vessels of the brain, suggesting that M-Ang2-EVs may have an inhibitory effect on brain-wide inflammation, indicating a direction for further research. Overall, our approach has beneficial therapeutic effects on both spinal cord injury and postinjury brain inflammation.

## Conclusion

In this study, we employed biotechnological methods to integrate Ang peptide into microglial cells, subsequently transforming them into M2 phenotype microglia with anti-inflammatory properties. This engineering ensured that extracellular vesicles derived from these cells retained anti-inflammatory efficacy and targeting specificity. Following this, we treated them with melatonin to obtain M-Ang2-EVs. These engineered EVs not only targeted the site of spinal cord injury but also promoted remyelination, axon growth, neuronal cell survival, repaired the blood-spinal cord barrier, and alleviated brain inflammation. These advancements collectively enhance neuroregeneration and repair processes postspinal cord injury, improving motor function and alleviating cognitive impairments in mice. This approach represents a promising new therapeutic strategy for spinal cord injury.

## Methods

### Cell Culture

Mouse microglial cell line (BV2) was obtained from the Shanghai Cell Research Center (Shanghai, China). Mouse brain microvascular endothelial cells (bEnd.3) and mouse cerebellar astrocytes (C8-D1A) were purchased from Procell Life Science & Technology Co., Ltd. (Wuhan, China). All cells were cultured in DMEM (4.5 g/L glucose) supplemented with 10% fetal bovine serum (FBS) and 1% penicillin/streptomycin in a 37 °C incubator with 5% CO2. To induce M2 polarization of BV2 microglial cells, the cells were treated with 20 ng/mL interleukin-4 (IL-4) for 48 h. The M2 phenotype of BV2 cells was confirmed using flow cytometry, immunostaining, and PCR analysis.

To mimic in vivo inflammatory responses, BV2 microglial cells were treated with lipopolysaccharide (LPS) at 1 μg/mL for 24 h, and C8-D1A astrocytes were treated with LPS at 100 ng/mL for 24 h. For the oxygen-glucose deprivation and reoxygenation (OGD/R) experiments, bEnd.3 cells were subjected to a glucose-free medium (Gibco) and incubated in a humidified anaerobic chamber (Don Whitley Scientific, UK) containing 5% CO2 and 95% N2 (oxygen concentration <0.2%) for 6 h. Postincubation, the cells were returned to the maintenance medium and placed back into a conventional incubator for 24 h to recover.

### Construction of Ang-Lamp2b Plasmid and Transfection

The Ang peptide (TFFYGGSRGKRNNFKTEEYC) was fused with the Ang-Lamp2b-EGFP fragment and inserted into the lentiviral vector plv-irs-puro. This vector, along with the packaging and envelope plasmids required for lentivirus construction, was transfected into HEK293T cells using Lipofectamine 2000 (Invitrogen, USA). The resulting viral particles were then used to infect BV2 cells. Following infection, the cells were selected using puromycin, ultimately yielding a BV2 cell line stably expressing the Ang-Lamp2b-EGFP fusion protein.

### Extraction and Characterization of EVs

M2-type microglial cells were obtained by treating BV2 cells expressing the Ang-Lamp2b-EGFP fusion protein with IL-4. These cells were then cultured in exosome-depleted medium for 24 h, and the supernatant was collected. EVs were extracted using ultracentrifugation. The collected supernatant was centrifuged at 300*g* for 10 min and 2000*g* for 10 min at 4 °C, followed by centrifugation at 10,000*g* for 20 min. The supernatant was filtered using a 0.22 μm sterile filter (Millipore). Subsequently, the filtered supernatant was centrifuged at 100,000*g* for 70 min using an ultracentrifuge. The pellet was resuspended in PBS and centrifuged again at 120,000*g* for 70 min. The final pellet was resuspended in PBS for further use.

The morphology of the EVs was observed using transmission electron microscopy (TEM). The diameter distribution of the EVs was analyzed using nanoparticle tracking analysis (NTA). The zeta potential of the EVs was measured, and the surface markers of the EVs were identified using Western blot analysis.

### EVs In Vitro Phagocytosis Assay

EVs were incubated in PBS containing 4 mg/mL Dil solution (Molecular Probe, USA) at a dilution of 1:200. The mixture was centrifuged at 100,000*g* for 70 min at 4 °C to remove excess dye. The pellet was resuspended in PBS, and this process was repeated three times. The Dil-labeled EVs were then cocultured with microglial cells, endothelial cells, and astrocytes for 24 h. Following coculture, the cells were fixed and observed to assess the uptake of EVs by each cell type.

### Flow Cytometry

Identification of M2-type Microglial Cells: M2-type microglial cells were washed with PBS and digested with EDTA-free trypsin (Gibco). The cells were washed three times and then incubated with CD11b antibody (Invitrogen) at 4 °C for 30 min. Following the manufacturer’s instructions, the cells were lysed and washed using the Adult Brain Dissociation Kit (Miltenyi). CD206 antibody (Invitrogen) was added to the cell suspension, and the cells were incubated at 4 °C for 30 min. The cells were then fixed, filtered through a cell strainer, and analyzed using a flow cytometer (Becton Dickinson).

Detection of Cell Apoptosis: microglial cells were collected as described above. After washing, the cells were incubated with the Annexin V-FITC/PI apoptosis detection kit (Vazyme) for 15 min according to the manufacturer’s instructions. Finally, the cells were analyzed for apoptosis using flow cytometry.

### RNA Sequencing and Analysis

RNA was extracted using the Exosome RNA Isolation Kit (Norgen) according to the manufacturer’s instructions. The quality of the RNA was assessed using the Bioanalyzer 2100 and RNA 6000 Nano LabChip Kit (Agilent, CA, USA). Small RNA libraries were prepared with approximately 10 ng of total RNA following the protocol of the TruSeq Small RNA Sample Prep Kit (Illumina, San Diego, CA, USA). Single-end sequencing (1 × 50 bp) was performed on the Illumina Hiseq2500 platform at LC-BIO (Hangzhou, China) following the supplier’s recommended protocols. Differentially expressed miRNA targets were predicted using two algorithms, TargetScan 7.2 and miRanda 3.3a. Common target genes predicted by both algorithms were visualized using VennDiagram. Functional enrichment analysis of the target genes was performed using GO (http://www.geneontology.org/) and KEGG (http://www.genome.jp/kegg/).

### Animal Model

Mice were housed in a 12 h light/12 h dark cycle with free access to food and water. The experimental animal protocol was approved by the Animal Care and Use Committee of the First Affiliated Hospital of Nanjing Medical University. Anesthetized mice were secured on an animal surgery table. After shaving, the skin and muscles of the back were incised to expose the vertebral lamina. A laminectomy was performed to expose the T10 spinal cord. The mice were then fixed using the Louisville Impactor System, with CO_2_ impact pressure set to 0.14 MPa, and the injury depth adjusted to 0.8 mm with laser guidance. After the impact, the skin was sutured. The mice were returned to their original housing conditions and their bladders were manually expressed daily to assist with urination.

### In Vivo Imaging

Dil-labeled EVs were injected via the tail vein of mice. The mice were then anesthetized, and an incision was made on the back to expose the spinal cord. Using an in vivo imaging system (IVIS) (PerkinElmer, Waltham, MA, USA), imaging was conducted at 3, 6, 9, and 12 h postinjection to observe the distribution of EVs in the spinal cord. The mice were then euthanized, and the spinal cord, brain, heart, liver, spleen, lungs, and kidneys were harvested to analyze and quantify the distribution of EVs in different organs across various groups.

To maintain EV distribution in vivo, EVs were injected immediately after spinal cord injury and then at 1 day, 3 days, and 6 days postinjury. On the seventh day, spinal cords were collected and sectioned to observe the uptake of EVs by microglia/macrophages, astrocytes, and endothelial cells.

### Behavioral Analysis

#### BMS Scoring

As previously described, the locomotor function of mice was assessed at 1, 3, 7, 14, 21, and 28 days postspinal cord injury using the Basso Mouse Scale (BMS). The hind limb motor function was observed and evaluated by two unbiased experts. The BMS scoring ranges from 0 (complete paralysis) to 9 (normal hind limb motor function). The final BMS score was calculated based on the average score given by the two experts.

#### Catwalk Analysis

Mice were placed on a glass walkway in complete darkness, with sideboards ensuring that only one mouse could pass through the channel at a time. The mice were encouraged to walk from left to right across the walkway. The pressure exerted by their paws caused the glass floor to emit green light, which was recorded by a high-speed camera positioned below. After the walking session, the Catwalk software was used to analyze various gait parameters and locomotor activity, allowing observation of hind limb motor recovery.

#### Electrophysiology

As previously described,^60^ 28 days postinjury, mice were anesthetized, and motor evoked potentials (MEPs) were recorded using an electrophysiological instrument. The stimulating electrode was placed at the cervicomedullary junction, and the recording electrode was placed on the gastrocnemius muscle of the hind limb. A single 10 mA stimulus was applied to the motor cortex, and the average MEP amplitude and latency were analyzed.

#### Y-Maze Task

We employed the Y-maze task to assess spatial recognition memory in mice. The Y-maze consisted of three arms of equal dimensions (30 cm × 10 cm × 20 cm each).

Exploration phase: mice were initially placed in one arm (Arm A) and allowed to freely explore another arm (Arm B) for 8 min.

Memory phase: subsequently, mice were returned to the starting arm (Arm A) and allowed to freely explore all three arms for 5 min.

The number of entries into each arm was recorded during this phase.

Spontaneous alternation: spontaneous alternation (%) was calculated as [(actual alternations)/(total arm entries −2)] × 100. Spontaneous alternation serves as an indicator of spatial recognition memory, where a higher percentage indicates better memory performance.

This task assesses the ability of mice to recognize and remember the spatial layout of the Y-maze, reflecting their spatial memory capabilities.

#### NOR (New Object Recognition) Test

As described previously,^59^ the NOR test is used to assess recognition memory in mice. The mice were asked to familiarize themselves with two identical objects in a 50 cm × 50 cm × 50 cm experimental setup. During the acquisition phase, the mice were allowed to freely explore the two objects for 10 min. During the familiarization phase, the mice were placed in the same experimental setup with the two objects for 10 min. The mice were then removed from the experimental setup every 10 min, and one of the familiar objects was replaced with a new object the mice were not used to. A mouse’s nose and/or front PAWS pointing to an object within 2 cm or touching was identified as exploratory behavior. The discriminant index calculates the percentage of time spent exploring the new object versus the total time spent on the two objects.

#### TS (Tail Suspension) Test

The TS test assesses depression-like behavior in mice, based on the observation that mice develop immobile postures when placed under hemodynamic stress that is inevitably suspended by the tail. As previously mentioned,^61,62^ TS was performed at week 10. Hang each mouse with tape at a height of 50 cm, 1 cm from the tip of the tail. The duration of inactivity was recorded throughout the 5 min test period. Static is defined as passive hanging and complete immobility.

### Immunofluorescence Staining

Tissue preparation: anesthetize the mouse and secure it on an animal surgical table. Open the thoracic cavity to expose the heart, cut open the right atrium, insert a scalp vein needle into the left ventricle, and fix it with a needle holder. Connect the other end to a perfusion machine. Begin perfusion with physiological saline to flush out the blood, then switch to perfusion with formaldehyde. The mouse’s tail will twitch and the body will stiffen during perfusion. Once the liver enlarges and turns pale, extract the spinal cord. After fixation and dehydration of the spinal cord tissue, embed it in paraffin and section it.

Cell and tissue staining: after deparaffinizing the spinal cord sections or fixing the cells, wash them with PBS and permeabilize with 0.3% Triton X-100. Block with an immunofluorescence blocking solution, ensuring the procedure is performed in the dark. Incubate overnight at 4 °C with the primary antibody. The next day, incubate with the corresponding fluorescent secondary antibody and stain the nuclei with DAPI. Observe the immunofluorescence staining under a fluorescence microscope.

### Single-Cell RNA-Seq Data Processing

RNA-sequencing data from individual cells was analyzed utilizing the Seurat package (version 4.3.0.1) in the R programming environment. The initial count matrices, derived from the 10X Genomics Cell Ranger pipeline, were imported and consolidated into a unified Seurat object. Cells were filtered out based on the following criteria: mitochondrial gene expression exceeding 20% of the total; erythrocytic gene (either Hbb or Hba) expression surpassing 5%; or a count of fewer than 200 unique genetic features. For each cell, gene expression values were normalized to the total transcript expression, multiplied by a factor of 10,000, and subsequently log-transformed. Normalization involved centering the values at the mean and scaling them relative to the standard deviation within each cell.

Principal component analysis (PCA) was conducted to achieve dimensionality reduction of the scaled data. Subsequently, dimensionality reduction was further achieved using Uniform Manifold Approximation and Projection (UMAP) and t-Distributed Stochastic Neighbor Embedding (t-SNE), focusing on the first 30 principal components. Hierarchical clustering was then applied to the UMAP-reduced data with 30 dimensions, employing Seurat’s built-in Louvain algorithm at a resolution parameter set to 0.5, thereby delineating a specific number of cellular clusters.

### Statistical Analysis

Statistical analysis is performed using GraphPad Prism 10.1.2 software (San Diego, CA, USA). Data comparisons between groups are conducted using Student’s *t*-test and one-way analysis of variance (ANOVA), followed by Tukey’s post hoc test for multiple comparisons. Significance levels are defined as *p < 0.05, **p < 0.01, ***p < 0.001. Each experiment is repeated at least three times, and results are expressed as mean ± standard error of the mean (SEM).

## Supporting Information Available

Enrichment analysis of TOP10 miRNAs in EVs; biosafety analysis of EVs, Ang2-EVs and M-Ang2-EVs; miRNAs difference analysis and enrichment analysis of Ang2-EVs and M-Ang2-EVs; volcano plot for gene differential analysis of cell populations and GO enrichment analysis of highly expressed genes in spinal cord tissue after M-Ang2-EVs treatment; identification of Neuron subtypes and OPC subtypes in the spinal cord after M-Ang2-EVs treatment; validation of miRNA expression in EVs; phagocytosis of M-Ang2-EVs by oligodendrocytes and neurons (PDF)

## Notes

Ethics approval and consent to participate: the Nanjing Medical University Ethics Committee approved the animal experiments for this study.

## Notes

Consent for publication: all the authors agree with the publication.
