# Supplementary material for: Engineered Extracellular Vesicles Modified by Angiopep-2 Peptide Promote Targeted Repair of Spinal Cord Injury and Brain Inflammation
Source: ACS Nano. 2025 Jan 24;19(4):4582–600. doi: 10.1021/acsnano.4c14675 (PMC11803916; doi:10.1021/acsnano.4c14675)
Supplement: Supplementary file 1 — nn4c14675_si_001.pdf [file nn4c14675_si_001.pdf]

Engineered extracellular vesicles modified by Angiopep-2 peptide promote targeted repair of spinal cord injury and brain inflammation

Guang Kong[2*], Jie Liu[3*], Juan Wang[4*], Xiaohu Yu[1], Cong Li[1], Mingyang Deng[1], Minhao Liu[1], Siming Wang[1], Chunming Tang[5#], Wu Xiong[1#], Jin Fan[1#]

[1]Department of Orthopedics, The First Affiliated Hospital of Nanjing Medical University, 300 Guangzhou Road, Nanjing (210000), Jiangsu, China.

[2]Department of Orthopedics, Xijing Hospital, Fourth Military Medical University, Xi'an (710000), Shaanxi, China.

[3]Department of Orthopedics, The Affiliated Taizhou People's Hospital of Nanjing Medical University, Taizhou School of Clinical Medicine, Nanjing Medical University, 366 Taihu Road, Taizhou (225300), Jiangsu, China.

[4]Department of human anatomy, School of Basic Medicine, Nanjing Medical University, Nanjing (210000), Jiangsu, China.

[5]Department of Pharmaceutics, School of Pharmacy, Nanjing Medical University, 300 Guangzhou Road, Nanjing (210000), Jiangsu, China.

[*] Guang Kong, Jie Liu, and Juan Wang contributed equally to this work.

[#]Corresponding Author:

Jin Fan Email: fanjin@njmu.edu.cn

Wu Xiong Email: a13707912710@163.com

Chunming Tang Email: cmtang@njmu.edu.cn

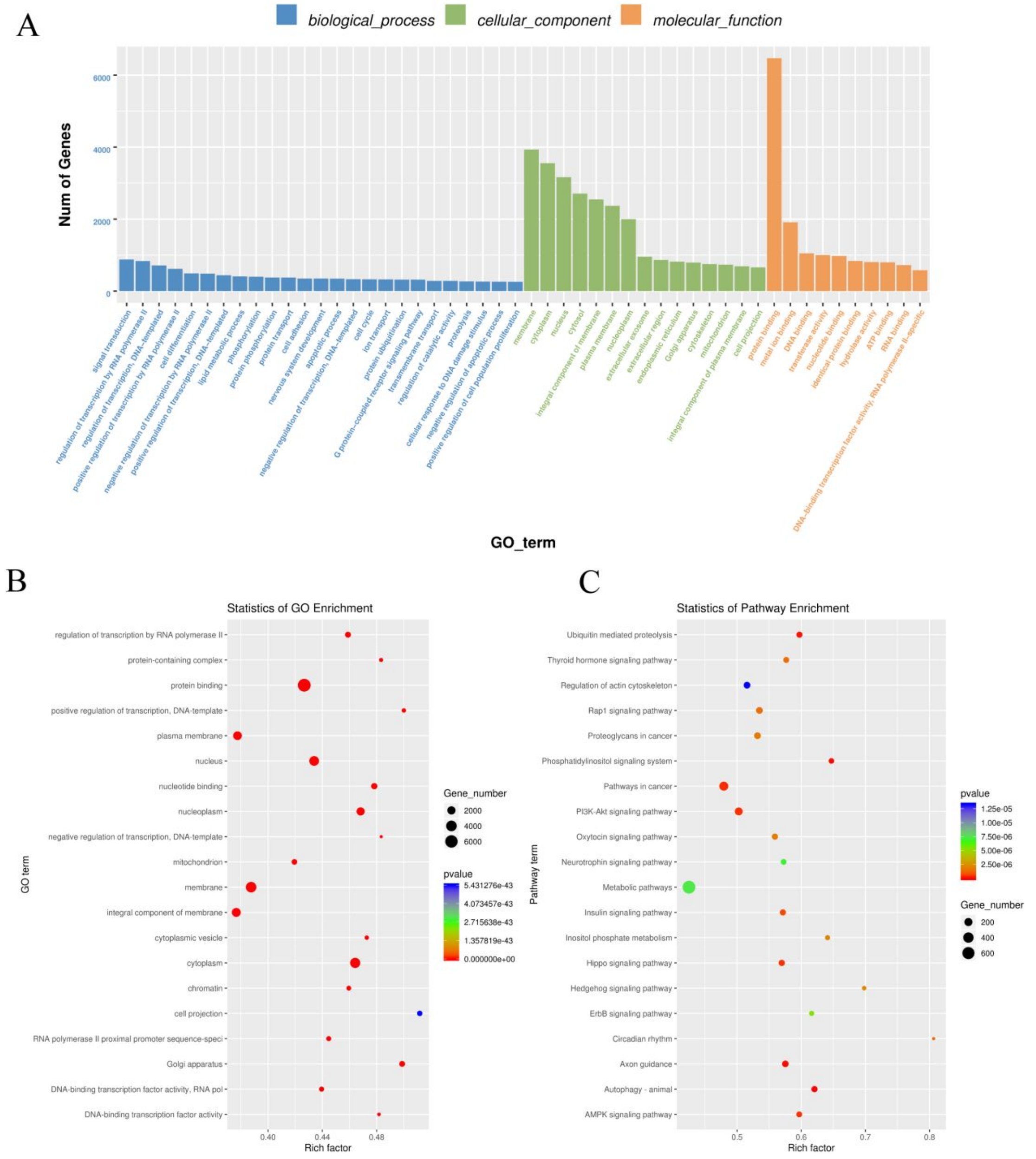

Figure. S1：Enrichment analysis of TOP10 miRNAs in EVs

A: biological process, cellular process, and molecular process enrichment analysis of TOP10 miRNAs in EVs. **B.** GO enrichment analysis of TOP10 miRNAs in EVs. **C:** Pathway enrichment analysis of TOP10 miRNAs in EVs.

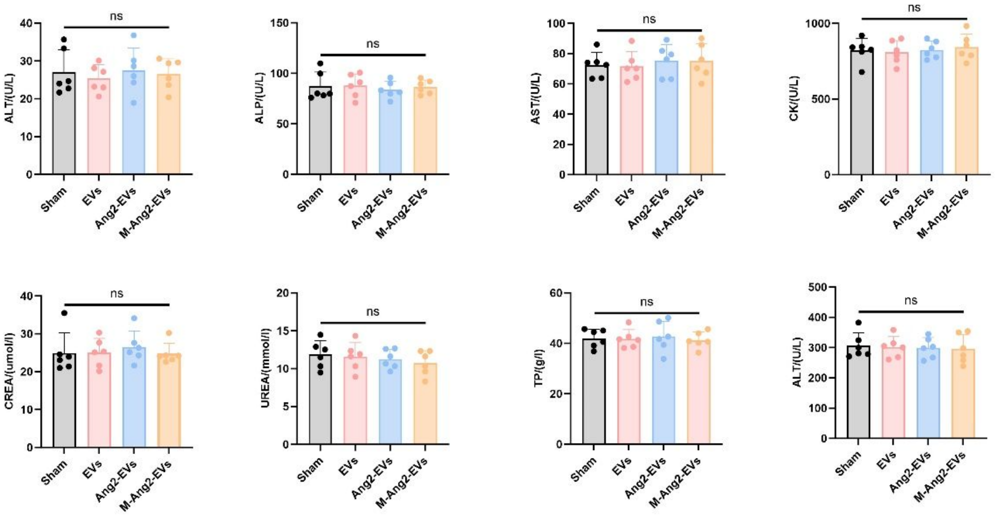

Figure. S2：Biosafety analysis of EVs, Ang2-EVs and M-Ang2-EVs

A: Quantification of blood biochemical indexes in mice after PBS, EVs, Ang2-EVs and M-Ang2-EVs treatment. n=6.

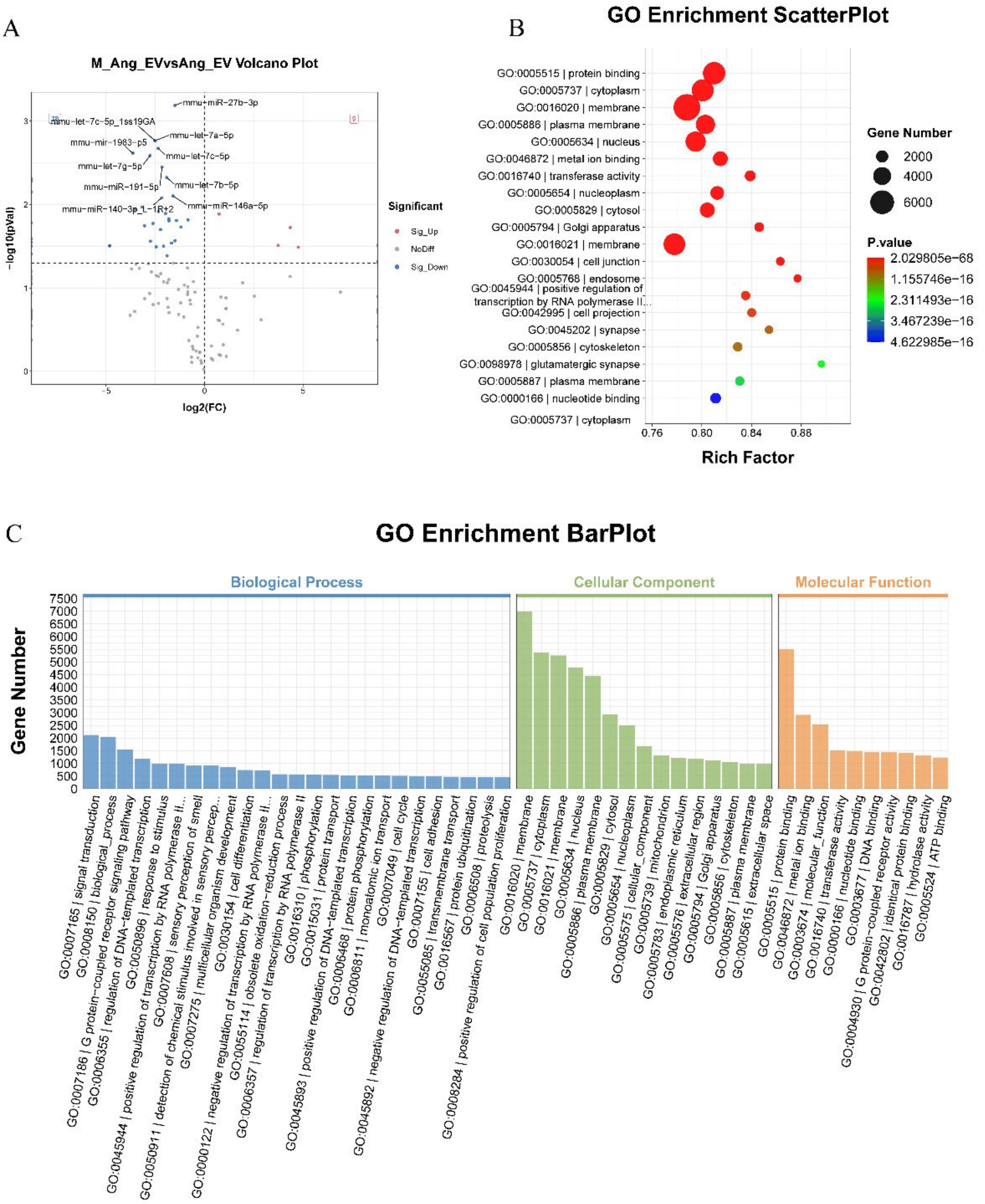

Figure. S3：miRNAs difference analysis and enrichment analysis of Ang2-EVs and M-Ang2-EVs

A: Volcanic maps of miRNA differences between Ang2-EVs and M-Ang2-EVs. **B:** GO enrichment scatterplot analysis of miRNA with high expression of M-Ang2-EVs. **C:** GO enrichment barplot analysis of miRNA with high expression of M-Ang2-EVs.

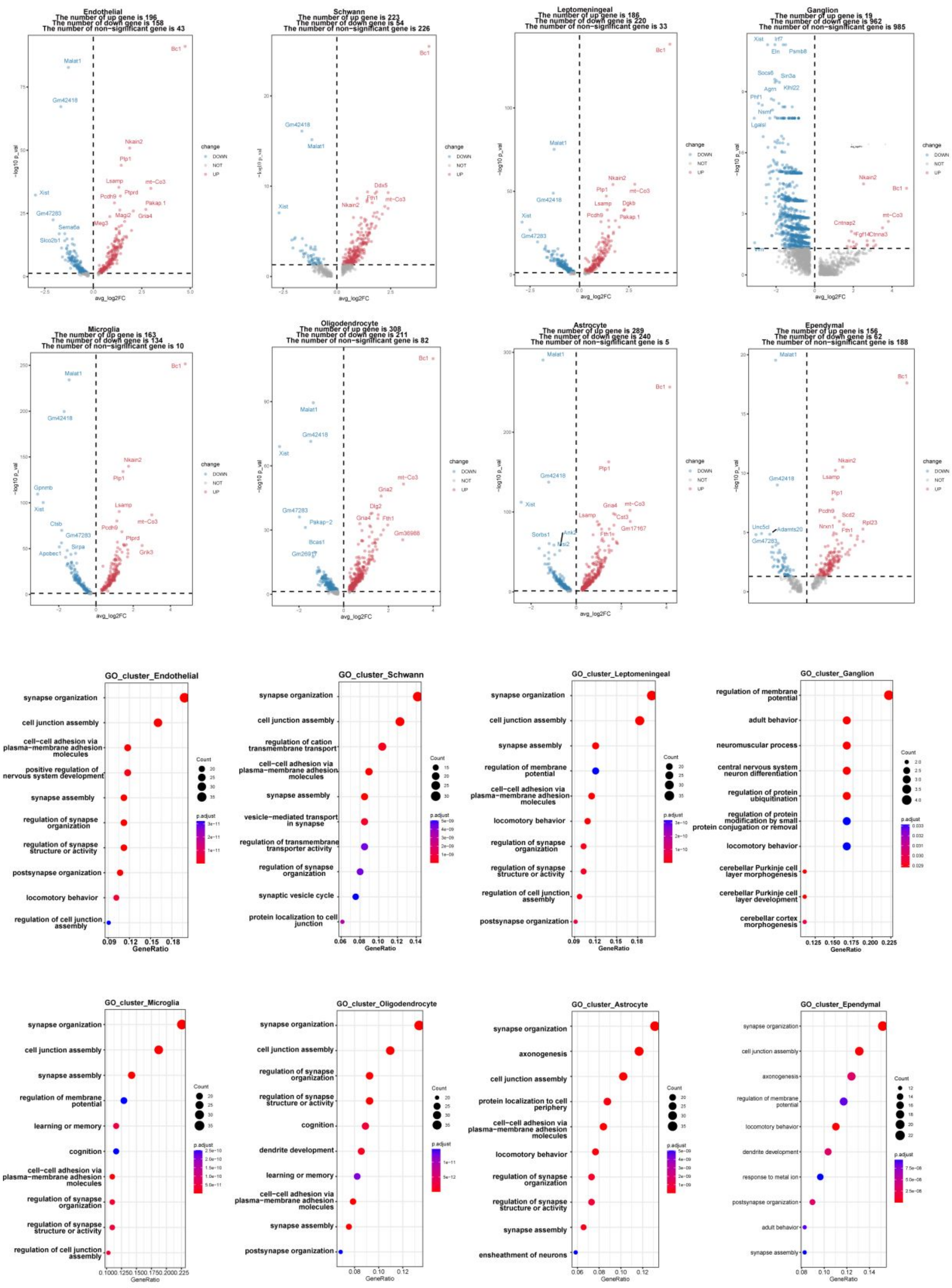

Figure. S4：Volcano plot for gene differential analysis of cell populations and GO enrichment analysis of highly expressed genes in spinal cord tissue after M-Ang2-EVs treatment

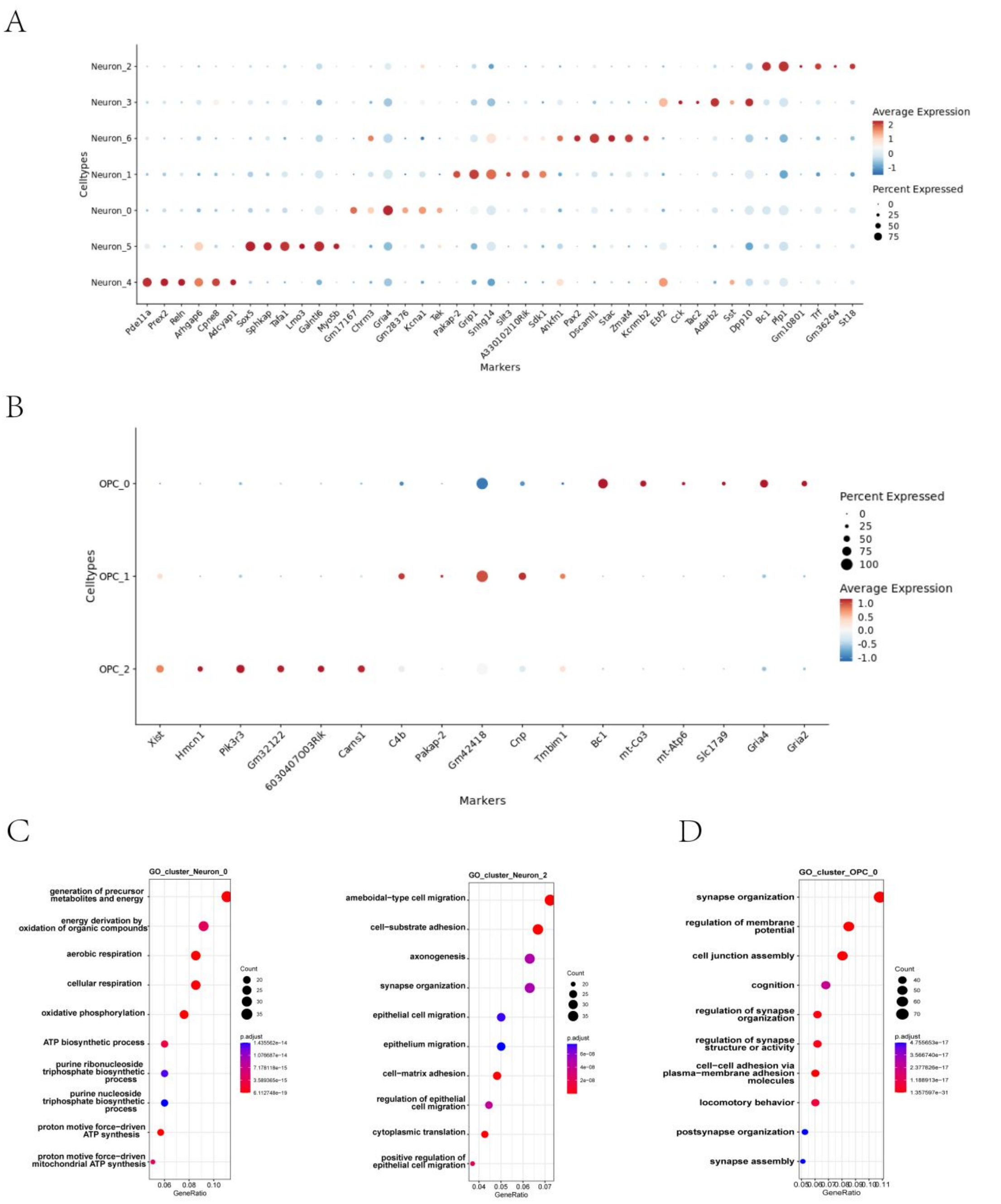

Figure. S5：Identification of Neuron subtypes and OPC subtypes in the spinal cord after M-Ang2-EVs treatment

A-B: Dot plot of the marker for each cluster. The color of the dot indicates the average RNA expression of the gene in the cell type, and the size of the dot indicates the percentage of cells in the cluster that express the gene. **C-D:** GO enrichment analysis revealed Neuron0, 2 and OPC 0 cell population functions.

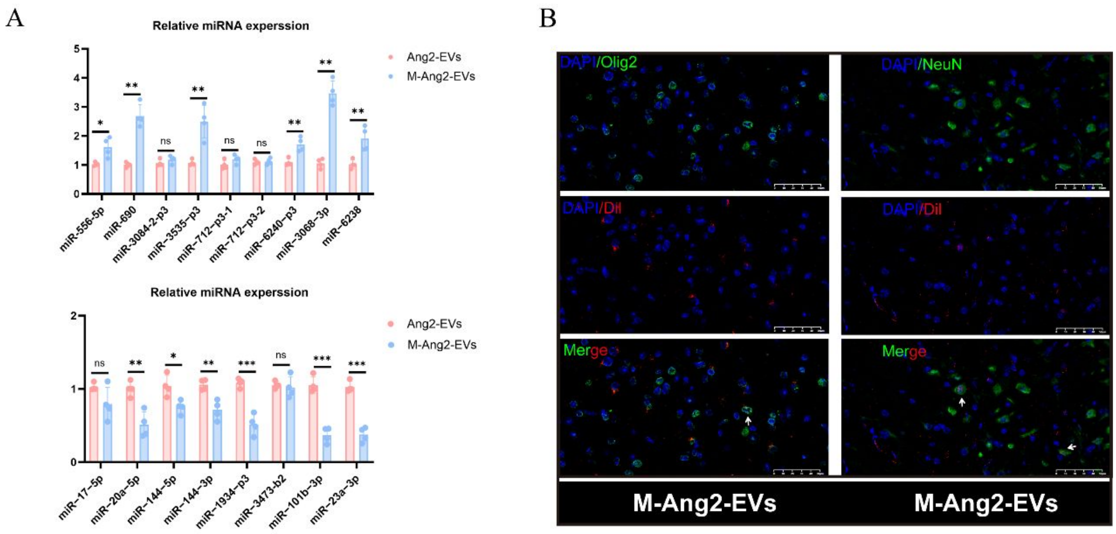

Figure. S6：**A:** qRT-PCR was used to detect the expression of miRNAs in the Ang2-EVs and M-Ang2-EVs groups. **B:** Immunofluorescence staining shows that a small number of oligodendrocytes (Olig2) and neurons (NeuN) have phagocytosed M-Ang2-EVs (arrows show co-location).
